# Gene—diet interactions in cardiovascular disease: insights from nutrigenetics, nutrigenomics, and precision nutrition

**DOI:** 10.3389/fnut.2026.1916937

**Published:** 2026-09-14

**Authors:** Mariam Ramadan, Amie Jobe, Ranjit Vijayan

**Affiliations:** 1 Department of Nutrition and Health, College of Medicine and Health Sciences, United Arab Emirates University, Al Ain, United Arab Emirates; 2 Department of Biology, College of Science, United Arab Emirates University, Al Ain, United Arab Emirates; 3 Zayed Bin Sultan Center for Health Sciences, United Arab Emirates University, Al Ain, United Arab Emirates; 4 Center for Excellence in Teaching and Learning, United Arab Emirates University, Al Ain, United Arab Emirates

**Keywords:** cardiovascular disease, gene-diet interaction, nutrigenetics, nutrigenomics, polygenic risk score, precision nutrition

## Abstract

Cardiovascular disease (CVD) remains the leading global cause of mortality, with diet as a major modifiable determinant of risk. However, substantial interindividual variability in lipid, glycemic, inflammatory, and blood pressure responses to dietary interventions suggests that uniform dietary recommendations may not optimally benefit all individuals. Nutrigenomics and nutrigenetics provide mechanistic and genetic frameworks to explain this heterogeneity by linking dietary exposures to gene expression, epigenetic remodeling, and genotype-dependent metabolic responses. In this context, this review synthesizes evidence on genome-wide interaction analyses, Mendelian randomization, and emerging multi-omics research. Across major dietary exposures, including fat, carbohydrates, sodium, and plant-derived bioactive compounds, genetic variation in pathways governing lipid transport and metabolism, glucose regulation, circadian biology, blood pressure control, and oxidative stress/repair has been associated with cardiometabolic responses. Mediterranean and low-fat/hypocaloric interventions demonstrate genotype-dependent changes in cardiometabolic biomarkers and, in some studies, transcriptomic signatures. Similarly, sodium sensitivity could be partly genetically influenced, supported by genome-wide interaction evidence, controlled sodium-feeding trials, and genetic causal inference. At the translational level, personalized nutrition trials indicate potential added benefit when dietary advice is genotype-informed, although reported benefits are generally incremental and often limited to intermediate cardiometabolic markers rather than validated cardiovascular outcomes. Generalizability is also constrained by methodological heterogeneity and limited population diversity. Accordingly, broader validation, standardization, and equitable implementation strategies are required before routine clinical adoption.

## 1 Introduction

Cardiovascular disease (CVD) remains the leading cause of morbidity and mortality worldwide, accounting for an estimated 19.8 million deaths in 2022, representing approximately 32% of all global deaths, with 85% attributable to heart attack and stroke (1). More than three-quarters of these deaths occur in low- and middle-income countries, reflecting persistent global health inequities and the growing cardiometabolic burden in resource-limited settings (1). CVD also accounts for at least 38% of premature deaths from non-communicable diseases occurring before the age of 70, underscoring its profound societal and economic impact (1).

CVD is a multifactorial condition resulting from complex interactions between genetic susceptibility, behavioral factors, and environmental exposures, including dietary transitions, urbanization, physical inactivity, obesity, tobacco use, and air pollution (1–6). Within this framework, diet represents a central and modifiable determinant of cardiovascular risk. Human dietary intervention studies indicate that selected dietary patterns can improve intermediate cardiometabolic risk markers, including body weight, lipid profiles, glycemic measures, blood pressure, and inflammatory markers, while some interventions also alter gut microbial composition and related metabolites (7–12).

In a large prospective UK Biobank cohort, participants with the highest vs. lowest adherence to a healthy, predominantly plant-based dietary pattern had an 8% lower incidence of CVD and 23% lower risk of CVD mortality (13). In contrast, high consumption of ultra-processed foods has been associated with an increased risk of cardiovascular events (14). Landmark interventions such as the Mediterranean and Dietary Approaches to Stop Hypertension (DASH) diets demonstrate clinically meaningful reductions in blood pressure and improvements in lipid profiles (7, 8, 15). However, considerable interindividual variability in dietary responses persists, with some individuals showing minimal improvement in low-density lipoprotein cholesterol (LDL-C) or other risk markers despite adherence (16). This heterogeneity highlights the need to understand the biological mechanisms underlying differential responses (Figure 1).

**Figure 1:**
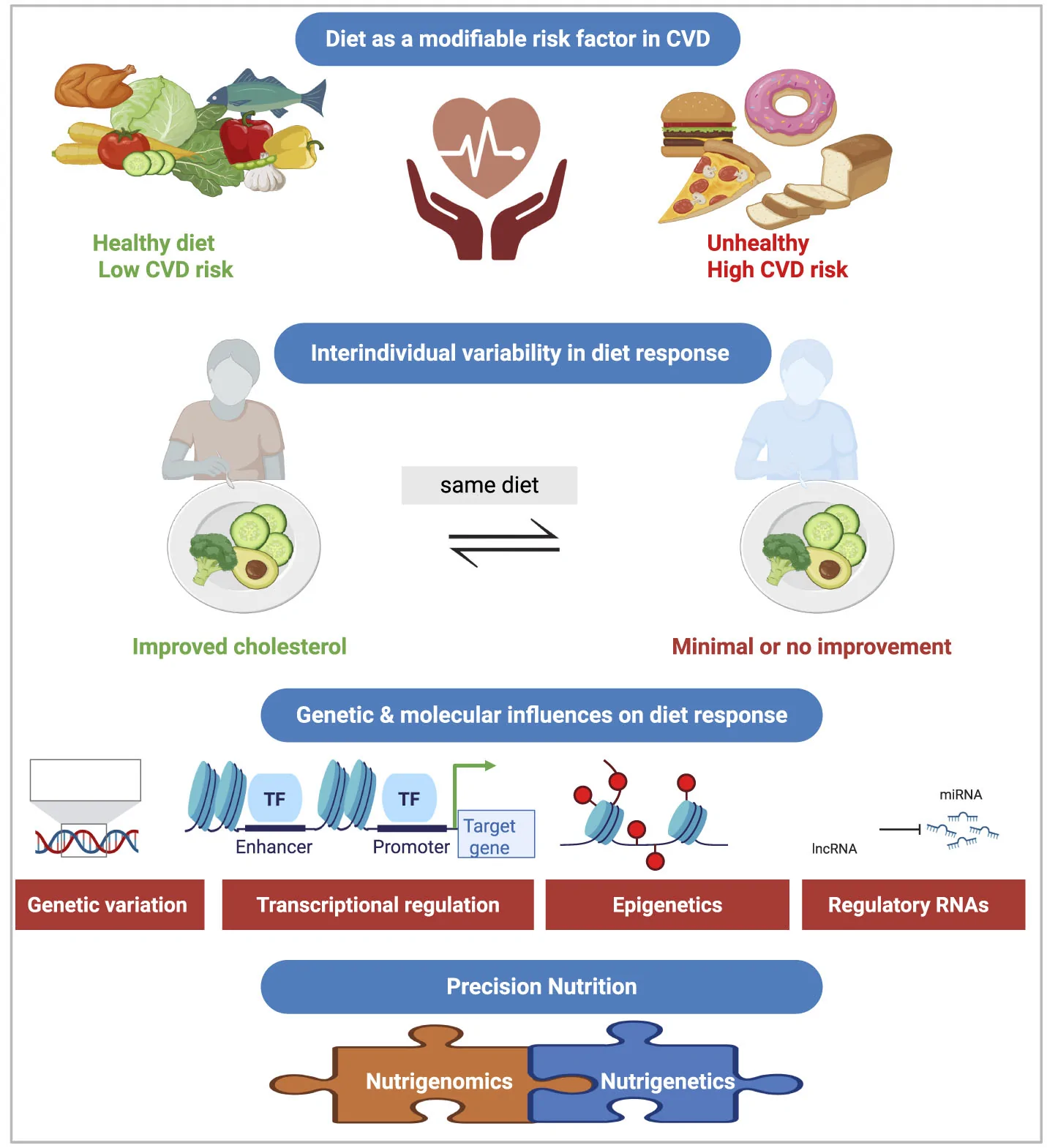
Interindividual variability in dietary response and the role of nutrigenomics in CVD. Diet influences cardiovascular disease risk, but responses vary among individuals. Genetic variation and molecular mechanisms may contribute to these differences, providing a foundation for precision nutrition through nutrigenomics and nutrigenetics. Created with BioRender.com.

Nutritional genomics encompasses two complementary fields: nutrigenetics and nutrigenomics. Nutrigenetics examines how inherited genetic variation contributes to interindividual differences in response to dietary components and modifies relationships between diet and disease (17, 18). In contrast, nutrigenomics examines how nutrients and bioactive food compounds influence gene expression and broader molecular responses involving the transcriptome, proteome, and metabolome (17, 18). Thus, nutrigenetics primarily addresses how genotype influences the response to diet, whereas nutrigenomics addresses how dietary exposures influence molecular function. Together, these complementary fields provide a framework for understanding why similar dietary exposures may produce different cardiometabolic responses among individuals.

At the molecular level, dietary components such as polyphenols and omega-3 fatty acids modulate inflammatory signaling, oxidative stress pathways, endothelial function, and lipid metabolism (12, 19, 20). Dietary exposures can also influence DNA methylation at metabolically relevant loci, suggesting a potential epigenetic pathway linking diet with cardiometabolic health (21–24). Human intervention studies have reported specific genotype-related differences in dietary response. *CLOCK* rs4580704 modified changes in high-sensitivity C-reactive protein and HDL/apolipoprotein A1 ratio following a low-fat diet (9). In PREDIMED, the same variant was associated with incident type 2 diabetes and cardiovascular events, although the genotype-by-Mediterranean-diet interaction for type 2 diabetes was borderline significant (25). In Food4Me, *APOE* ε*4* carriers showed greater reductions in saturated-fat intake following genotype-based advice, without corresponding genotype-specific improvements in the measured biomarkers (26).

Together, these findings form the scientific foundation for personalized nutrition, which seeks to tailor dietary recommendations according to genetic and molecular characteristics (27). Although translation into routine practice remains limited by methodological heterogeneity and incremental effects on intermediate cardiometabolic markers (28), growing evidence supports gene-diet interaction as a promising avenue for refining cardiovascular prevention strategies.

Against this background, this review synthesizes current evidence on gene-diet interactions in CVD, integrating nutrigenomics, nutrigenetics, and multi-omics research to highlight key variants, dietary exposures, and molecular mechanisms relevant to personalized cardiovascular nutrition.

## 2 Methods

This narrative review was informed by a structured literature search conducted in PubMed/MEDLINE, Scopus, and Google Scholar, with no lower date restriction; the final search was conducted in February 2026. Search terms were combined using Boolean operators and included: “cardiovascular disease,” “coronary artery disease,” “hypertension,” “dyslipidemia,” “cardiometabolic risk,” “nutrigenomics,” “nutrigenetics,” “gene-diet interaction,” “gene-nutrient interaction,” “precision nutrition,” and “personalized nutrition.” Additional searches included dietary and molecular terms relevant to the review, such as “dietary fat,” “omega-3 fatty acids,” “carbohydrate,” “glycemic response,” “sodium,” “salt sensitivity,” “Mediterranean diet,” “DASH diet,” “polyphenols,” “fiber,” “vitamin D,” “polygenic risk score,” “epigenomics,” “transcriptomics,” “metabolomics,” “microbiome,” and “multi-omics.” Reference lists of relevant articles were manually screened to identify additional studies.

The review primarily focused on human evidence. Eligible original studies included randomized controlled trials, controlled feeding studies, observational cohort and case-control studies, genome-wide gene-diet interaction analyses, Mendelian randomization studies, polygenic risk score analyses, and multi-omics investigations that examined relationships among dietary exposures, genetic variation, molecular responses, cardiometabolic risk markers, or cardiovascular outcomes. Reviews, meta-analyses, consensus statements, and selected mechanistic papers were used for background and interpretations, but original research articles formed the primary evidence base. Studies were excluded if they were not directly related to gene-diet interactions, nutrigenomics, nutrigenetics, precision nutrition, or cardiovascular/cardiometabolic outcomes; lacked sufficient methodological detail; reported duplicate findings; or were outside the scope of cardiovascular nutrition. Following screening and selection, 65 original research papers were included as the core evidence base.

In synthesizing the evidence, greater interpretive weight was given to findings supported by human randomized controlled trials, controlled feeding studies, prospective cohorts, Mendelian randomization analyses, genome-wide interaction studies with replication, and clinically meaningful cardiometabolic outcomes. Randomized controlled trials and controlled feeding studies were considered the strongest evidence for intervention-related effects, particularly when genotype-diet interactions were pre-specified or replicated. Mendelian randomization studies were interpreted as causal-influence evidence, while recognizing their dependence on instrumental-variable assumptions. Genome-wide interaction studies with independent replication were considered stronger evidence of association than single candidate-gene analyses. Prospective cohort, case-control, cross-sectional, candidate-gene, and secondary analyses were interpreted as observational or associative evidence and were not considered sufficient to establish causality. Multi-omics studies were interpreted primarily as mechanistic or biomarker evidence unless embedded within controlled interventions. Accordingly, causal language was reserved for randomized, controlled, or causal-inference designs, whereas observational findings were described as associations.

## 3 Nutrigenomics in CVD

### 3.1 Overview of nutrigenomic mechanisms in CVD

Nutrigenomics provides a mechanistic framework for understanding how dietary exposures regulate gene expression, epigenetic architecture, and molecular signaling pathways implicated in cardiovascular disease (29). Nutrients influence transcriptional programs in metabolically active tissues, including liver, adipose tissue, vascular endothelium, and immune cells, through modulation of transcription factors, nuclear receptors, and chromatin accessibility (30, 31). Genome-wide interaction studies and polygenic analyses suggest that dietary patterns such as low-fat, Mediterranean, and high-carbohydrate diets could elicit genotype-dependent molecular responses, highlighting multilayered diet-genome interactions [(32–35); Figure 2].

**Figure 2:**
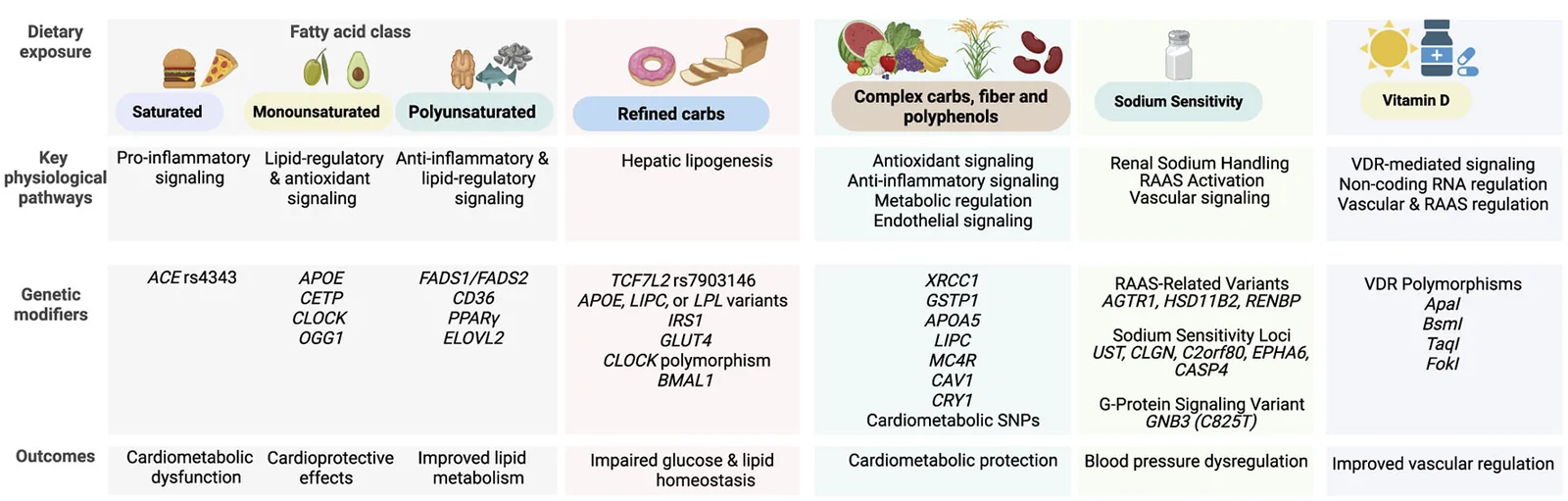
Nutrigenomic modulation of cardiometabolic pathways across dietary exposures. Dietary exposures regulate cardiometabolic pathways through specific molecular mechanisms. These effects are modified by genetic variation, resulting in interindividual differences in lipid metabolism, inflammation, glucose homeostasis, vascular function, and cardiovascular disease risk. Created with BioRender.com.

Dietary sodium represents another key nutrigenomic determinant of cardiovascular risk. Variability in blood pressure response to sodium intake is influenced by genetic variation in renal sodium transport, renin-angiotensin-aldosterone system (RAAS) components, and vascular signaling pathways (36–38). Evidence from genome-wide interaction analyses, controlled sodium-feeding trials, and Mendelian randomization supports a genetically mediated basis for sodium sensitivity, reinforcing sodium restriction as a potential precision nutrition target (37–45).

At the molecular level, inflammation is a central pathway linking diet and CVD. High-glycemic-index carbohydrates acutely increase NF-κB activation in peripheral blood mononuclear cells (46), whereas a saturated-fat-rich diet induces a pro-inflammatory gene-expression profile in adipose tissue (47). Human dietary studies also demonstrated genotype-dependent variation in selected cardiometabolic outcomes. In CORDIOPREV, *CLOCK* rs4580704 modified high-sensitivity C-reactive protein and HDL/apolipoprotein A1 responses to a low-fat diet (9), while *CETP* rs3764261 modified HDL-C and triglyceride responses to Mediterranean vs. low-fat diets (48). In PREDIMED, *OGG1 rs1052133* was associated with all-cause and cardiovascular mortality, with evidence of interaction with vegetable intake but not with Mediterranean-diet assignment (49). Hydroxytyrosol-rich extra-virgin olive oil and mango interventions reported changes in gene expression, including pathways related to oxidative stress (50, 51). In contrast, pomegranate extract produced medication-dependent changes in gut microbial composition and selected cardiometabolic biomarkers, with exploratory associations between genetic variants and microbial taxa (52). Fruit-and-vegetable interventions improved plasma antioxidant capacity, resistance to experimentally induced DNA damage, and retinal microvascular measures, with some responses varying according to genotype.

Epigenetic remodeling represents an additional layer (53). Mediterranean diet adherence is associated with differential DNA methylation in genes involved in inflammation and lipid metabolism (54). At the same time, weight-loss interventions modify methylation of fatty acid oxidation genes such as carnitine palmitoyltransferase 1A (*CPT1A*), paralleling cardiometabolic improvements (55). Non-coding RNA mechanisms may also contribute to diet-related cardiovascular responses. In patients with acute coronary syndrome, vitamin D supplementation was associated with reduced circulating levels of miR-361–5p, long non-coding RNA (lncRNA), maternally expressed three (*MEG3)*, and cardiac-hypertrophy-associated epigenetic regulator (lncRNA Chaer) (56).

Collectively, nutrigenomics integrates transcriptional, epigenetic, and regulatory RNA mechanisms to explain how dietary exposures influence lipid metabolism, glucose homeostasis, endothelial function, and immune activation, providing a molecular basis for precision cardiovascular nutrition (Figure 3 and Table 1).

**Figure 3:**
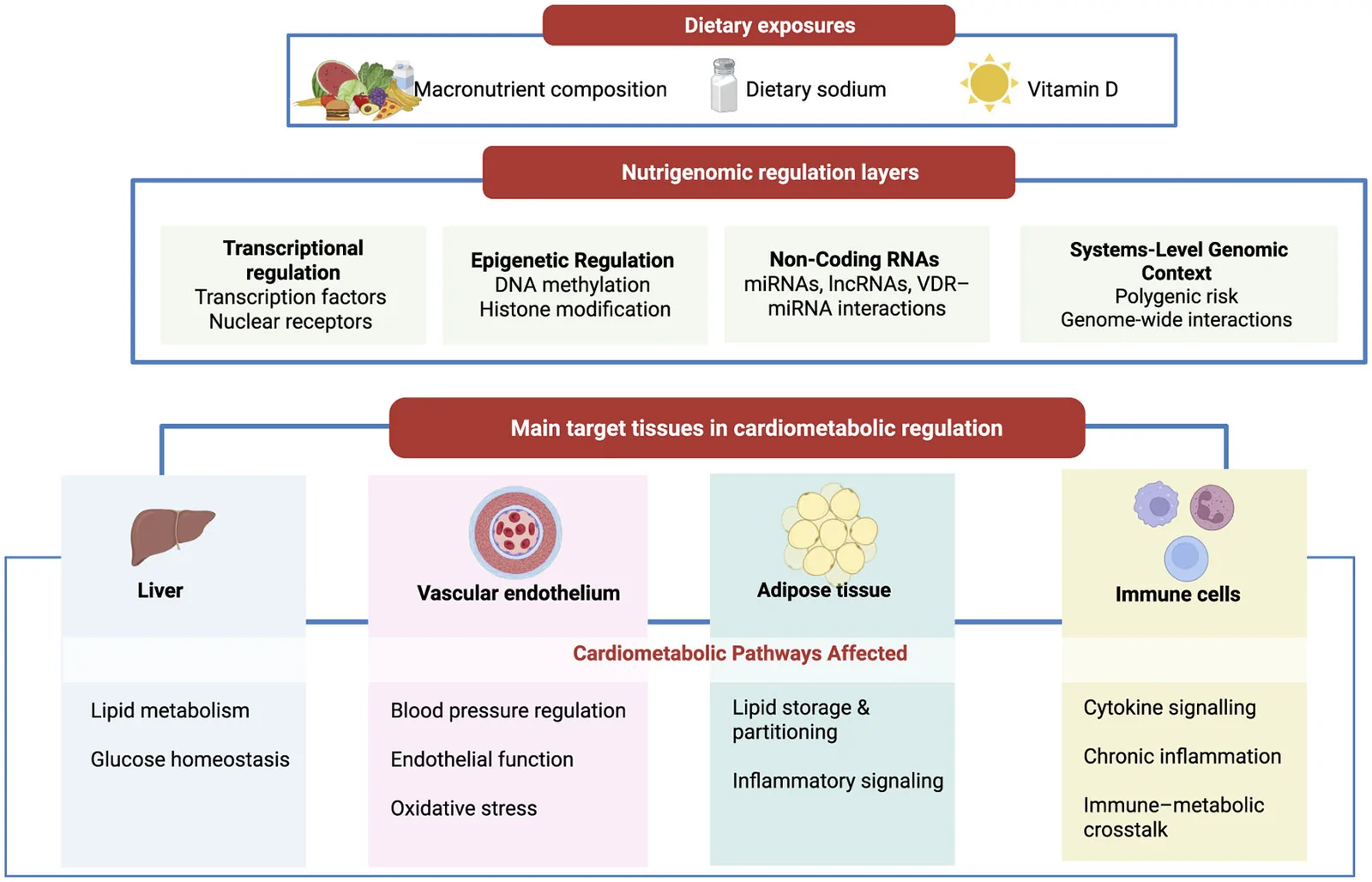
Nutrigenomic regulation of cardiometabolic pathways in CVD. Dietary factors influence cardiovascular disease through genetic and molecular mechanisms acting across key metabolic tissues, driving variability in cardiometabolic pathways. Created with BioRender.com.

**Table 1:** Major dietary exposures and gene-diet interactions influencing cardiovascular risk and metabolic outcomes.

Dietary exposure	Study design	Settings/countries	Genes, variants, or molecular features	Main findings	References
Mediterranean diet	RCTs and secondary RCT analyses; randomized crossover trial; prospective cohorts multi-omics study; epigenome wide analysis; nested case-control study	Spain, Israel, UK, US, Greece, and seven Eurpoean countries (Food4Me) cohorts	*CLOCK* rs4580704; CETP rs3764261; *OGG1* rs1052133; *ZPR1* rs964184; *TCF7L2* rs7903146; *MLXIPL* rs3812316; *PTGS2* rs20417 and *IL6* rs1800795; 300-SNP CVD PRS and multivariant GRS; cholesterol efflux-related expression involving A*BCA1, ABCG1, RXRA, RXRB, NR1H2, NR1H3*, and *PPARD*; Mediterranean-style-diet associated DNA-methylation signatures; fecal bile acids and microbial bile-acid-metabolism features	DIRECT-PLUS identified diet-related microbial and bile-acid changes associated with cardiometabolic responses (10). Mediterranean-diet interventions modified associations involving *CLOCK, CETP, ZPR1, TCF7L2*, and *MLXIPL*, although some formal interaction tests were borderline or non-significant (25, 48, 63, 64, 110). *OGG1* interacted with vegetable intake but not Mediterranean-diet assignment (49), while *PTGS2* and *IL6* did not modify inflammatory responses (111). Mediterranean interventions produced modest cholesterol-efflux-related expression changes without genotype stratification (29). Mediterranean-diet adherence was associated with DNA methylation and lower mortality or stroke risk, largely independently of aggregate genetic risk (35, 54, 102, 125). A synthesis of PREDIMED findings supports the overall cardiovascular benefits of Mediterranean-diet intervention and provides clinical context for the reported gene-diet associations (62).	(10, 25, 29, 35, 48, 49, 54, 62–64, 102, 110, 111, 125)
Low-fat and hypocaloric diets	RCTS and secondary trial analyses; genome-wide interaction analysis; weight-loss intervention	Spain and US	*CLOCK* rs4580704; *APOA1* rs670; 1, 760-variant gene-diet interaction score; 201-variant CAD PRS; *CPT1A* DNA methylation	In CORDIOPREV *CLOCK* rs4580704 modified hs-CRP and HDL/apolipoprotein A1 responses to a low-fat diet (9). A 1,760-variant score explained a proportion of the variability in 1 year LDL-C responses to dietary fat reduction (32). Diabetes prevention interventions improved cardiometabolic risk factors largely regardless of CAD PRS, with only nominal interaction evidence (34). Higher baseline *CPT1A* methylation predicted greater triglyceride and VLDL improvements during low-fat weight-loss diets (55). *APOA1* rs670 was associated with insulin-resistance and HDL-C responses to hypocaloric diets, although anthropometric improvements occurred across genotypes (82).	(9, 32, 34, 55, 82)
Dietary fat type/saturated fat replacement	Controlled sequential feeding; randomized crossover and parallel dietary trials; secondary intervention analyses	UK, Canada, Germany	*ACE* rs4343; *APOE* ε2/ε3/ε4 and rs1064725; LPL rs320 and rs328; *ABCA1* rs2066714; *ABCG5* rs6720173; *CYP7A1* rs3808607; *DHCR7* rs76024; multivariant lipid-related SNP combinations	Under high-fat or saturated-fat conditions, *ACE* rs4343 GG carriers demonstrated greater increase in ACE expression, circulating ACE systolic BP, and impaired glucose tolerance (59, 60). Combinations of lipid-related variants explained part of the variability in LDL-C and triglyceride responses to diets differing in fatty-acid composition (81). Replacing saturated fat with low-glycemic index carbohydrate produced greater total-cholesterol and lipoprotein B reductions in APOE ε4 carriers (72). In DIVAS *APOE* rs1064725 modified total cholesterol responses to MUFA replacement, whereas the examined *LPL* interactions were not significant (73).	(59, 60, 72, 73, 81)
Polyunsaturated fatty acids, omega-3/fish oil and vegetable oils	Population cohorts; genotype-selected intervention; RCTs and crossover trials; *post hoc* RCT analysis; supplementation study; epigenome-wide study; observational GWAS	China, Finland, US, Canada, UK, Iran	*CD36* rs1527483; *FADS1* rs174550, rs174556, and rs174561; *FADS2* rs1535 and rs174583; *ALOX5* promoter repeat variants; *ELOVL2* rs953413; *ELOVL5* rs2397142; *CETP* rs5882; *SCD* rs2234970; *PPARα* rs6008259; *LIPF* rs814628; GJB6-GJB2-GJA3 locus rs112803755; remnant-cholesterol GRS; genome-wide DNA methylation	*CD36* rs1527483 modified association of omega-3 and DHA status with HDL-C triglycerides, and gut microbial features (57). *FADS1* modified inflammatory and lipid responses to linoleic acid and vegetable oils (66, 120). In OMEGA-REMODEL, *FADS2* rs1535 modified cardiac-remodeling and biomarker responses to high-dose omega-3 treatment after myocardial infarction (67). Fish oil did not significantly reduce carotid-plaque prevalence overall, and the exploratory remnant-cholesterol GRS interaction did not meet the conventional FDR threshold (68). *ALOX5* variants modified selected fatty-acid and HDL-particle responses (69). Omega-3 supplementation altered leukocyte DNA methylation at cardiometabolic loci (70), while an observational GWAS identified fish-oil-lipid interaction loci including rs112803755 (71). A controlled DHA trial reduced triglycerides independently of the examined lipid-related variants (127).	(57, 66–71, 120, 127)
Trans fatty acids	Randomized double-blind parallel trial	Japan	*FTO* rs9939609, *ADRB3* rs4994	Supplementation providing approximately 1% of energy from trans fatty acids for 4 weeks did not significantly alter serum lipids, glucose-related outcomes, or responses according to the examined *FTO* and *ADRB3* variants. The finding concerns this relatively low dose and short intervention and should not be generalized to higher trans-fat intake.	(126)
Sodium and low-sodium salt	Sequential controlled-feeding study; GWAS and genome-wide interaction studies; prospective and cross-sectional cohorts; cohort-based case-control study; two-sample MR; randomized low-sodium-salt trial	China, Taiwan, Korea, Iran, European-ancestry GWAS cohorts	*AGTR1* rs4524238 and rs3772616; *HSD11B2* rs5479; *RENBP* rs1557501 and rs2269372; *UST* rs13211840; *CLGN* rs2567241; LOC105369882 rs11104632; *ACE* I/D polymorphism *GNB3* rs5443; *SCNN1B* rs239345; *TRPV1* rs224534, rs4790151, and rs8065080; ENaC genetic risk scores; sodium-related GWAS loci including *FGF5, ATP2B1, NT5C2, HLA-DQB1*, and *RNF213*; 20 urinary sodium-to-creatinine instruments; 94 salt-adding instruments	Controlled feeding identified *AGTR1, HSD11B2*, and *RENBP* variants associated with BP responses to low- or high-sodium intake (37). Genome-wide studies identified population specific hypertension loci and sodium-interaction loci affecting systolic or diastolic BP (38, 39). *ACE* and *GNB3* interactions with sodium intake were associated with hypertension risk in population studies (40, 41). Two MR studies linked genetically predicted urinary sodium or salt-adding behavior to selected cardiovascular endpoints; these constitute causal inference evidence dependent on MR assumptions rather than proof of clinical causality (42, 43). *SCNN1B* and *TRPV1* variants were associated with sodium intake or diastolic BP in a small cross-sectional study (44). A 3-year RCT found greater systolic BP reduction from low-sodium salt among participants with higher ENaC SBP genetic-risk scores, with no corresponding DBP interaction (45).	(37–45)
Polyphenol-rich foods, fruits, vegetables and plant sterols	Acute RCT; randomized placebo-controlled crossover trials; single-arm interventions; cross-sectional study; pilot nutrigenetic study	Italy, Spain, Netherlands, Canada, Iran, Brazil	Expression of *CAT, SOD1*, and *USF1* exploratory *GNB3* rs5443, *TCF7L2* rs7903146, and *LEPR* rs1137101-microbiota association; 15-variant antioxidant/DNA-repair panel; transcriptomic signatures; composite GRS containing *CAV1* rs3807992, *CRY1* rs2287161, and *MC4R* rs17782313; *APOE* rs429358, *LPL* rs12678919, *MVK* rs2287218, *APOA5* rs3135506, and *LIPC* rs1800588	Phenol-rich olive oil altered antioxidant-gene expression and reduced oxidized-LDL-related markers (50). Pomegranate extract produced medication dependent microbiota changes, with exploratory SNP-microbiota associations rather than transcriptomic effects (52). A fruit-and-vegetable blend improved resistance to experimentally induced DNA damage and retinal vascular dilation, but genotype effects were generally limited (88). Mango intake produced gene-expression signatures alongside cardiometabolic changes (51). Cross-sectional polyphenol-GRS associations could not establish causality (90). Small orange-juice and plant-sterol interventions reported genotype-related differences in lipid or BP responses, but subgroup sizes were limited (92, 94).	(50–52, 88, 90, 92, 94)
Vitamin D supplementation	Open-label randomized trial with follow-up; prospective supplementation study	Egypt and Tunisia	*VDR ApaI* rs7975232, *BsmI* rs1544410, *TaqI* rs731236, and *FoqI* rs2228570; miR-361–5p and IncRNAs MEG3 and Chaer; *GC* rs4588, *CYP2R1* rs10766197 and rs12794714; *CYP27B1* and *CYP24A1*	In patients with acute coronary syndrome, vitamin D supplementation was accompanied by changes in ventricular, biomarker, and regulatory-RNA outcomes; *VDR* variants showed prognostic associations but were not demonstrated to modify treatment efficacy (56). In adults with vitamin D deficiency, *GC* and *CYP2R1* variants were associated with differences in circulating 25-hydroxyvitamin D response to supplementation, without assessment of cardiovascular outcomes (98).	(56, 98)
Personalized nutrition, overall dietary patterns, and complex exposures	Web-based RCT; genome-wide interaction analysis; randomized genotype informed dietary trial; cross-sectional gene-diet-medication analysis; longitudinal cohort; multicomponent personalized nutrition RCT	Seven Eurpoean countries (Food4Me), UK, Mexico, Romania, US, China	*APOE* ε4 defined by rs429358 and rs7412; genome-wide aggregate Gxdiet effects; 11-variant lipid panel including *LPL* rs13702, *APOA1* rs670, and *LIPC* rs1800588; *CYP1A2* rs762551; ancestry-specific CVD/metabolic-syndrome GRS; *FTO* rs1121980 and gut microbial features	Food4Me genotype-informed advice reduced saturated-fat intake compared with population level advice but did not outperform non-gene-based personalization or produce different responses between *APOE* ε4 carriers and non-carriers (26). UK Biobank genome-wide analyses found small and largely unreplicated genotype-by-diet effects (33). A Mexican trial reported selected lipid and inflammatory changes during a nutrigenetic diet, but superiority over the standard diet was inconsistent (74). Medication-dependent associations between caffeine intake, *CYP1A2*, cholesterol, and glucose were cross-sectional (108). ARIC identified ancestry- and GRS-related heterogeneity in associations between dietary patterns and metabolic syndrome (114). A multicomponent intervention reported *FTO*-related differences in anthropometric and lipid responses, with microbiome findings remaining exploratory (119)	(26, 33, 74, 108, 114, 119)

### 3.2 Nutrigenomic effects of dietary fats

Dietary fats are central to nutrigenomic regulation due to their strong influence on lipid metabolism, inflammatory signaling, and vascular function. In a controlled-feeding study, a saturated-fat-rich diet increased the expression of genes involved in inflammatory and immune processes in adipose tissue compared with a monounsaturated-fat-rich diet (47). Additional genotype-specific associations have been reported for unsaturated fats. *CD36* rs1527483 modified associations of omega-3 PUFA and DHA status with HDL-C, triglycerides, and gut microbial features, with favorable associations observed primarily among GG carriers (57). In PREDIMED, the *LPL* rs13702 variant, located within a miR-410 target site, was associated with triglycerides and stroke responses that varied according to unsaturated-fat intake and Mediterranean-diet assignment (58). Under high-saturated-fat dietary conditions, angiotensin I converting enzyme (*ACE*) rs4343 was associated with higher ACE expression and systolic blood pressure, suggesting genotype-specific RAAS sensitivity (59, 60).

In high-risk adults, Mediterranean diet interventions were associated with mild upregulation of cholesterol-efflux-related genes, including retinoid X receptor alpha (*RXRA*), retinoid X receptor beta (*RXRB*), ATP binding cassette subfamily A member 1 (*ABCA1*), ATP binding cassette subfamily G member 1 (*ABCG1*), nuclear receptor subfamily 1 group H member 2 (*NR1H2*), and peroxisome proliferator activated receptor delta (*PPARD*) (29). Mediterranean diet trials indicate genotype-dependent improvements in HDL-C, triglycerides, oxidative stress, and inflammation among carriers of apolipoprotein E (*APOE*), cholesteryl ester transfer protein (*CETP*), clock circadian regulator (*CLOCK*), and 8-oxoguanine DNA glycosylase (*OGG1*) variants (9, 25, 48, 49, 61–64).

Polyunsaturated fatty acids (PUFAs), particularly omega-3 fatty acids eicosapentaenoic acid (EPA) and docosahexaenoic acid (DHA), can suppress NF-κB signaling, reduce triglyceride synthesis through downregulation of sterol regulatory element-binding transcription factor 1 (*SREBF1*), and enhance fatty acid oxidation (65). Human studies indicate that genetic modification of PUFA responses is specific to the fatty-acid exposure and outcome examined. In a linoleic-acid-enriched intervention, fatty acid desaturase 1 *FADS1* rs174550 modified changes in plasma fatty-acid composition, fasting glucose, high-sensitivity C-reactive protein, and lipid mediators (66). In a *post hoc* analysis of OMEGA-REMODEL, fatty acid desaturase 2 (*FADS2)* rs1535 modified cardiac remodeling and biomarker responses to high-dose omega-3 supplementation after acute myocardial infarction, with greater reductions in left ventricular end-systolic volume index, NT-proBNP, and galectin-3 observed among GG carriers (67). In patients with type 2 diabetes, marine omega-3 supplementation did not significantly reduce carotid-plaque prevalence overall, and an exploratory interaction involving a remnant-cholesterol genetic risk score did not meet the conventional false-discovery-rate threshold (68). In healthy adults of African ancestry, *ALOX5* promoter repeat variants modified erythrocyte omega-3 incorporation and selected triglyceride and HDL-particle responses to fish oil supplementation (69). Omega-3 supplementation has also been associated with genome-wide changes in leukocyte DNA methylation, at loci related to inflammatory, lipid-metabolic, and cardiovascular pathways (70). Separately, a genome-wide association study identified fish-oil-lipid interaction loci, including rs112803755 in the GJB6-GJB2-GJA3 region (71).

Cholesterol transport pathways are also genotype-sensitive. *CETP* and *APOE* polymorphisms influence high-density lipoprotein cholesterol (HDL-C) and LDL-C responses to SFA and MUFA composition (26, 48, 72, 73), and nutrigenetic interventions demonstrate greater lipid and inflammatory improvements when dietary advice aligns with genotype (74).

Overall, dietary fats exhibit distinct molecular signatures affecting lipid homeostasis, inflammation, and vascular health, with genetic variation shaping some interindividual differences in responses. These findings support the relevance of nutrigenomics for CVD prevention; however, genotype-guided dietary fat recommendations require further replication in diverse cohorts and validation in adequately powered intervention studies using clinically meaningful cardiometabolic outcomes (Table 1).

### 3.3 Carbohydrate and glycemic response pathways

Carbohydrates exert significant nutrigenomic effects on glucose homeostasis, insulin signaling, lipogenesis, and circadian metabolic regulation (75, 76). High-glycemic and high-fructose diets activate SREBP-1c and acetyl-CoA carboxylase 1 (ACC1), increasing hepatic lipogenesis and promoting hypertriglyceridemia and insulin resistance (77, 78). Genetic variation modifies these responses, positioning carbohydrates as a genotype-sensitive macronutrient.

Transcription factor 7-like 2 (*TCF7L2*) is a central regulator of insulin secretion and glucose metabolism. The rs7903146 risk allele has been associated with impaired insulin secretion and incretin effects and increased hepatic glucose production (79). In a randomized crossover trial, TT homozygotes had a less favorable triglyceride-rich lipoprotein profile after a low-fat diet than after a Mediterranean diet (64). Similarly, PREDIMED found that Mediterranean-diet adherence and intervention attenuated genotype-associated differences in fasting glucose and lipid concentrations and stroke incidence (80).

Macronutrient composition trials further demonstrate genotype-dependent carbohydrate responses. Variants in *APOE, LIPC*, and *LPL* modify lipid and glycemic adaptation to diets differing in carbohydrate proportion (72, 81, 82). Genes involved in insulin signaling and glucose transport, including insulin receptor substrate 1 (*IRS1*) and glucose transporter type 4 (*GLUT4*), are also influenced by habitual carbohydrate availability and macronutrient composition, potentially contributing to differences in glucose and insulin sensitivity (83, 84).

Circadian regulation adds another layer of complexity. Carbohydrate timing interacts with circadian genes such as *CLOCK* and basic helix-loop-helix ARNT-like 1 (*BMAL1*) (85, 86). Evidence from dietary intervention studies suggests that *CLOCK* variation may modify responses to overall dietary patterns rather than carbohydrate timing specifically; in the CORDIOPREV trial, *CLOCK* rs4580704 modified inflammatory and lipid responses to a low-fat diet (9). In PREDIMED, the association between this variant and lower incidence of type 2 diabetes appeared stronger in the Mediterranean-diet group, although the interaction was borderline statistically significant (25). Neither study directly assessed carbohydrate timing or circadian misalignment.

In contrast, high-quality carbohydrates, particularly fiber and polyphenol-rich foods, modulate inflammation, oxidative stress, and lipid handling through short-chain fatty acid (SCFA) production and epigenetic mechanisms (87). Human interventions confirm beneficial gene-expression changes and improved endothelial function following fruit- and vegetable-based diets (51, 52, 88), while epigenome-wide studies associate complex carbohydrate intake with favorable methylation patterns at cardiometabolic loci (54).

Overall, carbohydrate quality, quantity, and timing modulate multiple molecular pathways influencing CVD risk, with genetic background shaping some of these effects. These findings support the potential relevance of genotype-informed carbohydrate strategies in precision cardiometabolic nutrition; however, routine clinical application requires further validation in adequately powered, diverse intervention studies using standardized dietary assessment and clinically meaningful cardiometabolic outcomes (Table 1).

### 3.4 Salt, electrolytes, and gene regulation in blood pressure

Sodium intake is a major determinant of blood pressure (BP), vascular stiffness, and CVD (89). Nutrigenomics evidence suggests that genetic variation modulates BP responses to sodium exposure through coordinated effects on renal sodium handling, RAAS activation, vascular tone, and intracellular signaling pathways (37). These mechanisms underpin the concept of salt sensitivity as a biologically heterogeneous and genetically influenced phenotype.

Large-scale genetic analyses support a causal role for sodium-related behaviors in cardiovascular outcomes. A two-sample Mendelian randomization analysis using 94 genetic variants associated with the frequency of adding salt to food found evidence consistent with higher risks of venous thromboembolism, atrial fibrillation and flutter, ischemic stroke, and peripheral artery disease (43). Separately, an analysis using 20 genetic instruments for urinary sodium-to-creatinine ratio reported associations with seven cardiovascular endpoints, including coronary atherosclerosis, ischemic heart disease, myocardial infarction, and peripheral artery disease. Because these findings depend on the validity of the genetic instruments and other Mendelian randomization assumptions, they should be interpreted as causal-inference evidence rather than definitive proof of clinical causality (42).

Genome-wide interaction analyses further demonstrate interindividual heterogeneity in sodium sensitivity. In the GenSalt study, variants in uronyl 2-sulfotransferase (*UST*), calmegin (*CLGN*), chromosome 2 open reading frame 80 (*C2orf80*), ephrin type-A receptor 6 (*EPHA6*), caspase 4 (*CASP4*), and related loci significantly modified systolic and diastolic BP responses to sodium intake (39), with replication in the MESA cohort confirming these interactions. These loci are involved in vascular remodeling, endothelial signaling, and renal sodium transport, emphasizing the polygenic architecture of sodium sensitivity.

In 1,906 Han Chinese adults, polymorphisms in RAAS-related genes, including angiotensin II receptor type 1 (*AGTR1*), hydroxysteroid 11-beta dehydrogenase 2 (*HSD11B2*), and renin binding protein (*RENBP*), altered BP responses during sequential low- and high-sodium diets (37). Carriers of specific AGTR1 alleles exhibited exaggerated BP elevation under high sodium exposures, consistent with enhanced angiotensin II signaling (37). In addition, population-based analyses demonstrate that interactions between ACE genotype and salt intake influence hypertension risk, with genotype-specific differences in BP responses depending on sodium exposure (40).

Additional support arises from G-protein signaling pathways. In a 10-year Taiwanese cohort, individuals with the Guanine nucleotide-binding protein beta-3 subunit (*GNB3*) rs5443 TT genotype who consumed more than 4,800 mg/day of sodium had an increased risk of hypertension, whereas this risk was attenuated at lower sodium intake levels (41).

Emerging evidence also suggests that genetic variation may influence sodium intake behavior itself. Variants in genes encoding epithelial sodium channel subunits (ENaC), including *SCNN1A, SCNN1B*, and *SCNN1G*, and transient receptor potential cation channel subfamily V member 1 (*TRPV1*) genes have been associated with differences in salt taste perception, sodium consumption, and blood pressure, indicating that genetic regulation of sensory pathways may contribute to interindividual variability in dietary sodium exposure and hypertension risk (44). Furthermore, large population-based analyses show that genetic susceptibility to hypertension differs according to sodium intake levels, with distinct loci associated with hypertension under low- vs. high-sodium conditions (38).

Together, evidence from Mendelian randomization, genome-wide interaction studies, cohort analyses, and controlled feeding trials suggests that sodium sensitivity is a phenotype that is partly genetically influenced and governed by integrated RAAS, renal, vascular, and neurohormonal pathways (37–45). These findings support sodium reduction as a promising precision nutrition target in genetically susceptible individuals, although prospective trials are still needed before genotype-guided sodium recommendations can be routinely implemented (Table 1).

### 3.5 Polyphenols, fiber, and antioxidant pathways

Polyphenols, fiber, and other plant-derived bioactive compounds have been associated with changes in oxidative stress, inflammation, endothelial function, and lipid metabolism, key pathways in CVD (90). These compounds regulate gene expression through coordinated modulation of Nrf2 antioxidant signaling, NF-κB inflammatory pathways, and epigenetic mechanisms, with responses varying by genotype (91).

Cross-sectional evidence suggests that genetic background may modify the association between dietary polyphenol intake and cardiometabolic markers. Among overweight and obese women, these associations varied across categories of a composite genetic risk score comprising melanocortin 4 receptor (*MC4R*), caveolin 1 (*CAV1*), and cryptochrome circadian regulator 1 (*CRY1*) variants (90).

Human intervention studies demonstrate diverse molecular and cardiometabolic responses to plant-derived bioactive compounds. Extra-virgin olive oil upregulates catalase (*CAT*), superoxide dismutase 1 (*SOD1*), and upstream transcription factor 1 (*USF1*) (50). Pomegranate extract produced medication-dependent changes in gut microbial composition; inflammatory and metabolic biomarkers and exploratory SNP-microbiota associations were also evaluated (52). In a randomized crossover MiBlend study, increased intake of fresh fruit-and-vegetable blends improved retinal microvascular dilation and resistance to experimentally induced DNA damage. Genotype-stratified analyses showed that wild-type carriers of *XRCC1* rs25487 experienced greater protection against DNA damage after the flavonoid-rich interventions, whereas wild-type carriers of *GSTP1* rs1695 showed greater retinal microvascular improvement after the fruit-and-vegetable interventions, particularly the polyphenol-rich blends; these differences remained significant after false-discovery-rate correction (88). Associations involving *HNF1A* rs1169288 and plasma antioxidant capacity were exploratory and did not remain significant after multiple testing (88). An 8-week mango intervention produced gene-expression signatures alongside changes in cardiometabolic outcomes (51). Separately, a pilot orange-juice intervention reported variation in lipid and blood-pressure responses according to *APOE, APOA1*, and LPL polymorphisms but did not evaluate gene expression (92).

Fiber-rich diets extend these effects through short-chain fatty acid production, AMPK activation, and downregulation of SREBP-1c-mediated lipogenesis (93). In a small randomized double-blind crossover trial, *APOA5* rs3135506 was associated with differences in both total cholesterol and LDL-C responses to plant sterol supplementation (94).

In a randomized, placebo-controlled trial, a grape extract supplement containing resveratrol reduced oxidized LDL and apolipoprotein B concentrations in patients undergoing primary cardiovascular prevention (95).

Collectively, human intervention studies show that polyphenols and fiber regulate molecular pathways central to CVD prevention, with responses shaped by genetic variation in antioxidant, lipid, and inflammatory genes. These findings support the integration of plant-based bioactives into precision cardiovascular nutrition strategies; however, the clinical translation of genotype-specific responses remains limited by heterogeneity in dietary assessment, intervention composition, sample size, and outcome selection, requiring further validation in larger and more diverse human studies (Table 1).

### 3.6 Vitamin D signaling, MicroRNAs, and long non-coding RNAs

Vitamin D contributes to cardiovascular regulation through effects on inflammation, vascular remodeling, calcium homeostasis, and immune signaling (96). Genetic variation in vitamin D transport, metabolism, and receptor pathways, together with downstream non-coding RNA networks, has been investigated as a potential source of heterogeneity in vitamin D status and cardiometabolic outcomes. However, evidence that vitamin D receptor (VDR) genotype modifies the cardiovascular efficacy of vitamin D supplementation remains limited (97, 98).

At the human nutrigenomic level, vitamin D supplementation has been associated with changes in miRNAs and lncRNAs implicated in cardiac fibrosis and remodeling (56). In a randomized study of patients with acute coronary syndrome, adding vitamin D to standard therapy was accompanied by changes in echocardiographic parameters, reductions in cardiac fibrosis markers, procollagen type III N-terminal peptide and soluble ST2, and lower circulating levels of miR-361–5p and the lncRNAs MEG3 and Chaer (56). In a separate prognostic analysis, *VDR BsmI* (rs1544410) and *FokI* (rs2228570) genotypes were associated with subsequent major adverse cardiovascular events, whereas *TaqI* (rs731236) was associated with heart failure. The study did not demonstrate a formal genotype-by-treatment interaction; therefore, these genetic findings should be interpreted as prognostic associations rather than evidence of genotype-specific supplementation efficacy (56).

Other studies further support the role of vitamin D-related pathways in modifying diet-induced cardiometabolic risk. For example, polymorphisms in genes involved in vitamin D transport and activation, including *GC* rs4588 and *CYP2R1* rs10766197 and rs12794714, were associated with differences in serum 25-hydroxyvitamin D responses following supplementation (98). Individuals carrying risk alleles exhibited reduced increases in circulating vitamin D levels, indicating that genetic background contributes to interindividual variability in supplementation efficacy and may influence downstream metabolic and cardiovascular pathways (98).

Beyond ncRNA modulation, vitamin D influences RAAS activity, endothelial nitric oxide synthesis, and immune cell gene expression, pathways that interface with dietary fat, carbohydrate, and polyphenol-driven mechanisms (99–101). These interconnected mechanisms underscore the relevance of vitamin D pathways within the broader nutrigenomic landscape of CVD.

Taken together, vitamin D-related pathways provide a biologically plausible link between nutrition, gene regulation, vascular function, inflammation, and blood pressure control. Nevertheless, genotype-specific cardiovascular responses to vitamin D remain incompletely established, and future studies should clarify whether variation in *VDR, GC, CYP2R1*, and related pathways predicts clinically meaningful benefit from supplementation or dietary intervention (Table 1).

### 3.7 Multi-omics integration in nutrigenomics

Systems-level approaches increasingly illustrate the complexity of gene-diet interactions by integrating genomics, transcriptomics, epigenomics, metabolomics, and metagenomics. These multi-omics frameworks provide insight into how dietary exposures interact with host genetic architecture and microbial functional capacity to shape cardiometabolic pathways in a genotype-dependent manner.

Genome-wide interaction and polygenic-score studies have investigated whether common genetic variation contributes to heterogeneity in cardiometabolic responses to diet, although findings have not been consistent. In the Women's Health Initiative, a gene-diet interaction score derived from 1,760 SNPs was associated with variability in LDL-C response to a dietary fat-reduction intervention (32). Most variants were intergenic or intronic, with CUB and sushi multiple domains 1 (*CSMD1)*, protein tyrosine phosphatase receptor type D (*PTPRD)*, and regulator of G protein signaling 12 (*RGS12)* containing the largest numbers of annotated variants. However, these genes were not individually established as modifiers of LDL-C responses to dietary fat (32). By contrast, a UK Biobank GxEsum analysis encompassing 12 cardiometabolic traits and 20 dietary exposures found that no diet-trait pair met the Bonferroni-corrected significance threshold. Genome-wide interaction effects explained a mean of only 0.1% of trait variance, compared with 16.8% explained by genetic main effects, indicating a limited detectable contribution of gene-diet interactions in this population (33). Polygenic risk score (PRS) studies have also examined genetic modification of associations with Mediterranean diet adherence, lifestyle or low-calorie interventions, and overall diet quality, but the reported interaction evidence has generally been limited or inconsistent (34, 35, 102).

Epigenomic evidence adds another regulatory layer. Mediterranean diet adherence is associated with differential methylation at cardiometabolic loci, and weight-loss interventions induce methylation changes at genes such as *CPT1A* that predict triglyceride and glucose responses (54, 55). Omega-3 supplementation has also been reported to induce DNA-methylation changes in blood leukocytes (70). Together, these findings suggest that diet-related epigenetic remodeling may represent a regulatory mechanism linking nutrition with cardiometabolic outcomes.

Transcriptomic analyses further demonstrate nutrigenomic plasticity. Human interventions involving hydroxytyrosol and mango consumption produced changes in gene-expression profiles alongside cardiometabolic outcomes (50, 51).

Metagenomics integration introduces a microbial dimension to gene-diet relationships. In a population-based cohort of 5,959 genotyped individuals with matched gut metagenomes and dietary records, 567 independent SNP-taxon associations were identified. Associations between variants at the LCT locus and several microbial taxa, including *Bifidobacterium*, differed according to dairy intake, illustrating how dietary exposure may modify host genetic associations with gut microbial composition (103). However, the direct implications of these findings for cardiovascular outcomes remain to be established.

Cardiovascular-specific multi-omics studies provide complementary evidence. In 1,241 middle-aged Europeans spanning healthy status, dysmetabolic conditions, and different stages of ischemic heart disease, integrated phenomic, gut metagenomic, and serum and urine metabolomic profiling identified alterations across the cardiometabolic disease spectrum. Approximately 75% of the microbiome and metabolome features distinguishing ischemic heart disease from healthy status were already present in individuals with dysmetabolism after adjustment for medication and lifestyle factors, suggesting that these alterations may precede clinically apparent disease. Selected ischemic-heart-disease-specific features also improved discrimination beyond conventional risk markers (104).

Cardiovascular-specific human studies have identified gut microbial functional pathways associated with atherosclerotic CVD, circulating lipids, and cardiovascular-health measures, including pathways involved in trimethylamine, lipopolysaccharide, short-chain fatty acid, and cholesterol metabolism. Paired metagenomic and metabolomic profiling has additionally identified cholesterol-transforming gut bacteria associated with lower fecal and plasma cholesterol concentrations (105, 106).

Diet-microbiome interactions also modify the association between Mediterranean diet and cardiometabolic risk. Long-term Mediterranean diet adherence reshapes microbial pathways involved in fiber degradation and short-chain fatty acid production. Yet, its cardioprotective association is significantly modified by specific taxa, including *Prevotella copri* (107). This illustrates that dietary benefits depend on both host genomic background and microbial functional context.

Cross-sectional evidence also suggests potential genotype-diet-medication interplay in xenobiotic metabolism. Among 358 adults, associations of habitual caffeine intake with total cholesterol and fasting glucose differed according to *CYP1A2* rs762551 genotype and concurrent medication use (108). The positive association between caffeine intake and total cholesterol was strongest among AA carriers not receiving statins and was attenuated with statin therapy. Caffeine intake was positively associated with fasting glucose among CC carriers not receiving oral antidiabetic medication but inversely associated with glucose among AA carriers receiving such treatment; no meaningful associations were detected among AC carriers (108). Because caffeine exposure was self-reported and no intervention was administered, these findings cannot establish causality or genotype-specific treatment efficacy.

Collectively, these systems-level findings indicate that cardiovascular risk emerges from coordinated interactions among host genetics, epigenetic regulation, microbial functional capacity, and diet-derived metabolites (Figure 4). Integrating metagenomics into nutrigenomic frameworks provides a more comprehensive biological explanation for interindividual variability in dietary response and reinforces the necessity of multi-omics approaches in advancing precision cardiovascular nutrition (Table 1).

**Figure 4:**
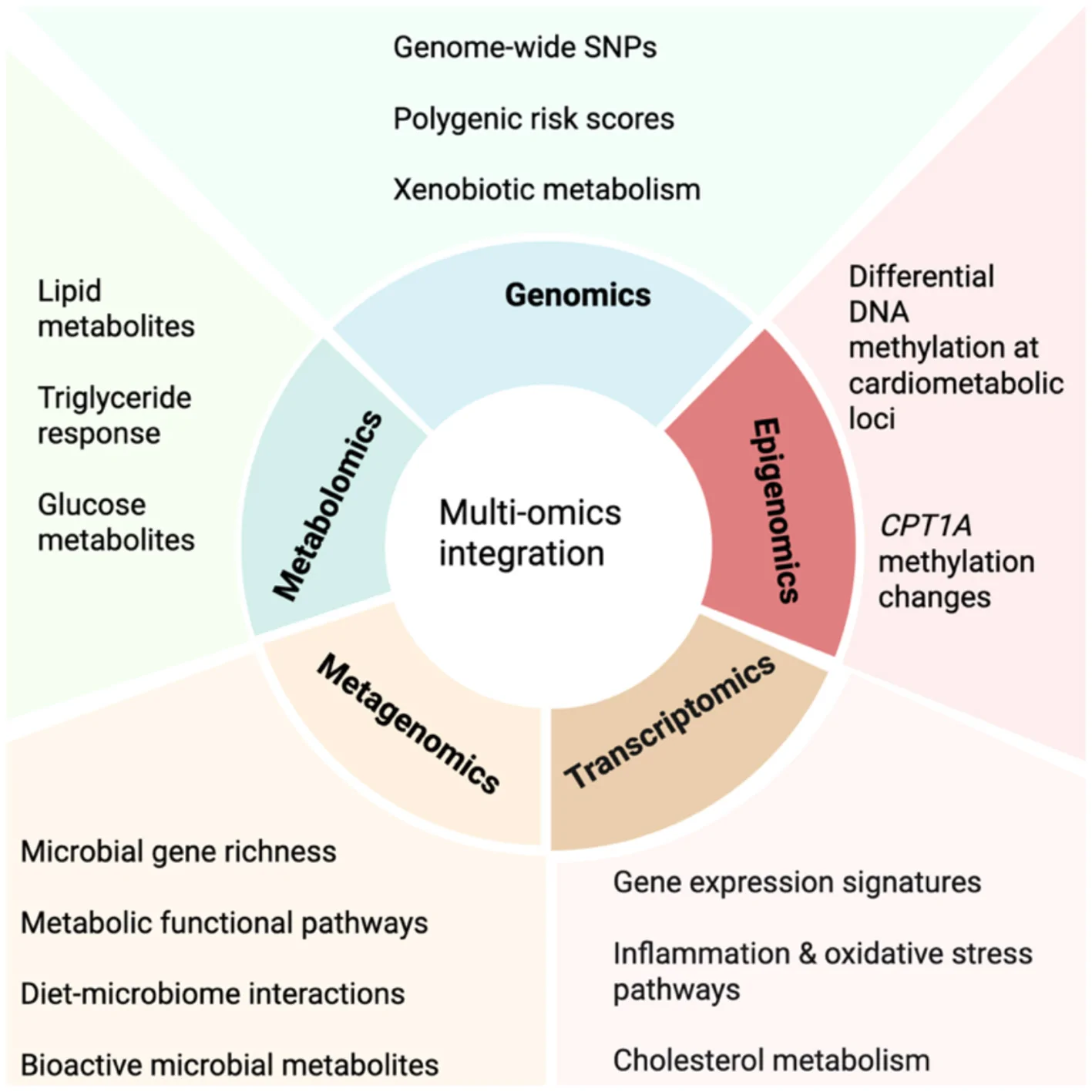
Systems-level multi-omics regulation of cardiometabolic pathways in nutrigenomics. Multi-omics approaches integrate genomic, epigenomic, transcriptomic, metabolomic, and metagenomic data to elucidate how dietary exposures interact with host genetic architecture and microbial functional capacity. Created with BioRender.com.

## 4 Dietary patterns and gene-diet interactions

### 4.1 Mediterranean diet and gene-diet interaction in cardiovascular disease

The Mediterranean diet represents one of the most extensively investigated models for examining gene-diet interactions in CVD. Characterized by high intake of extra-virgin olive oil, fruits, vegetables, legumes, whole grains, nuts, and moderate fish consumption, this dietary pattern has been evaluated in large cardiovascular-prevention trials. In the reanalyzed PREDIMED trial, the incidence of major cardiovascular events was lower among participants assigned to Mediterranean diets supplemented with extra-virgin olive oil or nuts than among those assigned to a reduced-fat control diet (109). However, accumulating evidence indicates that these benefits vary according to genetic background.

Large randomized controlled trials provide support for genotype-dependent responses. In the PREDIMED (PREvención con DIeta MEDiterránea) trial, variants in genes related to lipid metabolism, inflammation, and glucose regulation, including MLX interacting protein (*MLXIPL)*, lipoprotein lipase (*LPL*), FTO alpha-ketoglutarate dependent dioxygenase (*FTO*), melanocortin 4 receptor (*MC4R*), prostaglandin-endoperoxide synthase 2 (*PTGS2)*, glucokinase regulator (*GCKR*), and serpin family E member 1 (*SERPINE1)*, modified cardiometabolic responses to Mediterranean diets enriched with extra-virgin olive oil or nuts compared with low-fat control diets (62, 110). Similarly, in the Coronary diet intervention with olive oil and cardiovascular prevention (CORDIOPREV) study, polymorphisms in circadian genes such as *CLOCK* (rs1801260, rs3749474, and rs4580704) significantly influenced lipid, inflammatory, and metabolic responses over 12 months (9), underscoring the role of circadian regulation in dietary responsiveness.

Beyond analyses of individual polymorphisms, Mediterranean-diet interventions have also been evaluated at the transcriptomic level. In high-cardiovascular-risk subsamples from PREDIMED and PREDIMED-Plus, 12-month Mediterranean-diet interventions produced mild changes in the expression of cholesterol-efflux-related genes, including *ABCA1, ABCG1, RXRA, RXRB, NR1H2, NR1H3*, and *PPARD* (29). In PREDIMED, selected expression changes differed between the Mediterranean-diet and low-fat control groups; however, PREDIMED-Plus showed no significant between-group differences, and the study did not stratify responses by genotype (29). Separately, *CETP* (rs3764261) modified HDL-C and triglyceride responses to Mediterranean vs. low-fat diets (48). *OGG1* (rs1052133) was associated with all-cause and cardiovascular mortality, with evidence that its associations varied according to vegetable intake but not randomized Mediterranean-diet assignment (49).

Multi-omics and nutrigenetic analyses extend these findings. In the 18-month Dietary Intervention Randomized Controlled Trial Polyphenols Unprocessed (DIRECT-PLUS) trial, integration of host-genetic, gut-metagenomic, and metabolomic information improved the prediction and characterization of individual responses to polyphenol-enriched Mediterranean-diet interventions (61). Separately, *ZPR1* (rs964184) modified lipid responses in patients with coronary heart disease (63). In a randomized crossover study, *TCF7L2* (rs7903146) was associated with genotype-stratified differences in triglyceride-rich lipoprotein profiles following low-fat and Mediterranean diets, although the formal genotype-by-diet interaction was not statistically significant (64).

Not all studies demonstrate strong gene-diet interactions within Mediterranean dietary interventions. For instance, polymorphisms in inflammatory genes such as (*PTGS2 or COX2* rs20417) 765G>C and interleukin 6 (*IL6* rs1800795) 174G>C were associated with baseline inflammatory marker levels but did not significantly modify the anti-inflammatory response to a Mediterranean diet supplemented with virgin olive oil or nuts. Reductions in inflammatory biomarkers occurred regardless of genotype, suggesting that the cardioprotective effects of Mediterranean dietary patterns may occur independently of certain genetic variants (111).

Overall, evidence from randomized trials, cohort analyses, and transcriptomic studies indicates that Mediterranean diet responses can be influenced by genetic variation across pathways governing lipid metabolism, circadian biology, oxidative stress, and glucose homeostasis. However, some studies report limited or absent gene-diet interactions, indicating that the cardioprotective effects of the Mediterranean diet may occur broadly across genetic backgrounds. These findings position the Mediterranean diet as a central framework for precision cardiovascular nutrition, while highlighting the need for further research to clarify which genetic contexts meaningfully modify dietary response (Table 2).

**Table 2:** Genetic pathways mediating dietary responses in cardiovascular disease prevention.

Gene/variant or pathway group	Study designs and settings	Dietary exposures	Main gene-diet findings	References
Circadian related genes-*CLOCK* rs4580704; composite GRS containing *CRY1* rs2287161	RCT secondary analyses and cross-sectional study; Spain and Iran	Low-fat and Mediterranean diets; dietary polyphenols	In CORDIOPREV *CLOCK* rs4580704 modified hs-CRP and HDL/apolipoprotein A1 responses to a low-fat diet (9). In PREDIMED, the association between this variant and lower type 2 diabetes incidence was stronger in the Mediterranean-diet groups, although the formal interaction was borderline (25). Associations between polyphenol intake and cardiometabolic markers varied across a composite GRS containing *CRY1, CAV1* and *MC4R* variants; effects cannot be attributed independently to *CRY1* (90).	(9, 25, 90)
Lipid transport and lipoprotein metabolism-*CETP* rs3764261, *APOE* variants, *LPL* rs13702*, APOA1* rs670, *LIPC* rs1800588, *APOA5* rs3135506, *ZPR1* rs964184*, MLXIPL* rs3812316, lipid-related multivariant panels; expression of *ABCA1, ABCG1, RXRA, RXRB, NR1H2, NR1H3, PPARD*	RCTs and secondary analyses; crossover trials; pilot interventions; Spain, UK, Canada, US, Mexico, and Brazil	Mediterranean and low-fat diets; saturated fat replacement; hypocaloric and genotype-informed diets; plant sterols; orange juice	*CETP, LPL, ZPR1*, and *MLXIPL* variants modified selected HDL-C, triglyceride, postprandial-lipoprotein, or myocardial-infarction outcomes during Mediterranean-diet interventions (48, 58, 63, 110). APOE-related responses differed when saturated fat was replaced with low-glycemic index carbohydrate or MUFA, whereas Food4Me found no differential responses between APOE ε4 carriers and non-carriers (26, 72, 73). *APOA1, LIPC*, and *APOA5* variants were associated with selected lipid responses to hypocaloric, genotype-informed, orange-juice, or plant sterol interventions, although several analyses were small or exploratory (74, 82, 92, 94). Mediterranean interventions produced modest changes in cholesterol-efflux-related gene expression without genotype stratification (29). Multivariant lipid panels explained part of the heterogeneity in responses to dietary fatty-acid composition (81).	(26, 29, 48, 58, 63, 72–74, 81, 82, 92, 94, 110)
Fatty acid metabolism, desaturation, elongation and handling-*CD36* rs1527483*; FADS1* rs174550, rs174556, and rs174561; *FADS2* rs1535 and rs174583, *ALOX5* promoter variants*; ELOVL2* rs953413, *PPARα* rs6008259; GJB6-GJB2-GJA3 locus rs112803755; remnant-cholesterol GRS	Cohorts; genotype-selected intervention; RCTs and crossover trials; *post hoc* RCT analysis; observational GWAS; China, Finland, US, Canada, UK, and Iran	Omega-3/fish oil/and DHA; linoleic-acid-enriched diet; sesame and canola oils	*CD36* rs1527483 modified association of omega-3 and DHA status with HDL-C, triglycerides, and gut microbial features (57). *FADS1* variants modified fatty-acid, inflammatory or lipid responses to linoleic acid and vegetable oils (66, 120). In OMEGA-REMODEL, *FADS2* rs1535 modified cardiac-remodeling and biomarker responses to high dose omega-3 supplementation after myocardial infarction (67). *ALOX5* promoter variants modified selected erythrocyte fatty-acid and HDL-particle responses to fish oil (69). A fish-oil GWAS identified lipid interaction loci, including rs112803755 (71). Marine omega-3 supplementation did not significantly reduce carotid-plaque prevalence overall, and an exploratory remnant-cholesterol GRS interaction did not meet the conventional FDR threshold (68). In a controlled DHA trial triglyceride reduction was not modified by the examined lipid metabolism variants (127).	(57, 66–69, 71, 120, 127)
Blood pressure, sodium sensitivity and RAAS-pathways-*AGTR1* rs4524238 and rs3772616; *HSD11B2* rs5479; *RENBP* rs1557501 and rs2269372; *ACE* I/D and rs4343; *GNB3* rs5443; *UST* rs13211840; *CLGN* rs2567241; LOC105369882 rs11104632; *SCNN1B* rs239345; *TRPV1* rs224534, rs4790151, and rs8065080; ENaC GRS; sodium-related GWAS and MR instruments	Controlled feeding; prospective and cross-sectional cohorts; genome-wide interaction studies; case-control analysis; two-sample MR; RCT; China, Taiwan, Korea, Iran, Germany, and European ancestry GWAS cohorts	Sequential low-and high-sodium diets; habitual sodium and salt intake; low-sodium salt; high-saturated-fat diet	Controlled feeding identified *AGTR1, HSD11B2*, and *RENBP* variants associated with BP responses to sodium manipulation (37). genome-wide analyses identified sodium-dependent hypertension and BP loci, including UST and CLGN (38, 39). Population studies reported interactions of sodium intake with *ACE* and *GNB3* in relation to hypertension, although these findings were observational (40, 41). *SCNN1B* and *TRPV1* variants were associated with sodium consumption or diastolic BP in a small cross-sectional study (44). A low-sodium-salt RCT found greater systolic BP reduction among participants with higher genetic-risk scores, without a DBP interaction (45). Sodium-related MR studies reported associations with selected cardiovascular endpoints but remain dependent on instrumental variable assumptions (42, 43). Separately, *ACE* rs4343 modified ACE, BP, and glucose responses under high-fat or saturated-fat conditions (59, 60)	(37–45, 59, 60)
Inflammation, oxidative stress, antioxidant defense, and DNA repair-*OGG1* rs1052133*; PTGS2* rs20417*; IL6* rs1800795; exploratory *HNF1A* effects; expression of *CAT, SOD1*, and *USF1;* transcriptomic signatures; composite GRS containing *CAV1* rs3807992	RCTs and secondary analyses; crossover and single-arm interventions; cross-sectional study; Spain, Italy, Netherlands, Canada, and Iran	Mediterranean diet; extra-virgin olive oil; fruit and vegetable blends; pomegranate extract; mango dietary polyphenols	*OGG1* rs1052133 was associated with mortality and interacted with vegetable intake, but not with Mediterranean-diet assignment (49). *PTGS2* and *IL6* variants were associated with baseline inflammatory markers but did not modify the anti-inflammatory response to Mediterranean diets (111). Phenol-rich olive oil changes antioxidant-related gene expression and oxidative-stress markers (50), while mango intake produced heterogeneous gene-expression signatures (51). A fruit-and-vegetable blend improved DNA damage resistance and retinal vascular dilation but genotype effects were generally limited apart from an exploratory *HNF1A* findings (88). Pomegranate extract produced medication-dependent microbiome and biomarker changes rather than transcriptomic effects (52). Polyphenol associations varied across a composite *CAV1*-*CRY1*-*MC4R* GRS preventing attribution to an individual variant (90).	(49–52, 88, 90, 111)
Glucose regulation and carbohydrate response pathways-*TCF7L2* rs7903146, *ACE* rs4343; *APOA1* rs670; composite GRS containing *MC4R* rs17782313	Randomized crossover and parallel dietary trials; cohort and secondary trial analyses; Spain, Germany, US and Iran	Mediterranean diet and low-fat diets; high-fat diet; hypocaloric diets; dietary polyphenols	Mediterranean-diet intervention attenuated some adverse cardiometabolic and stroke associations of *TCF7L2* rs7903146 (80). A randomized crossover trial found genotype-stratified differences in triglyceride-rich lipoprotein profiles after low-fat and Mediterranean diets, although the formal genotype-by-diet interaction was not significant (64). Under high-fat conditions, *ACE* rs4343 GG carriers demonstrated greater deterioration in glucose tolerance (59). *APOA1* rs670 was associated with insulin-resistance and HDL-C responses during hypocaloric diets (82). Associations involving *MC4R* were evaluated as part of a composite polyphenol-related GRS and cannot be interpreted as an isolated *MC4R* effect (90).	(59, 64, 80, 82, 90)
Vitamin D signaling and metabolism-*VDR ApaI* rs7975232, *BsmI* rs1544410, *TaqI* rs731236, and *FokI* rs2228570; *GC* rs4588; *CYP2R1* rs10766197 and rs12794714; *CYP27B1* and *CYP24A1* variants; miR-361–5p and IncRNAs MEG3 and Chaer	Open-label randomized trial with follow-up and prospective supplementation study; Egypt and Tunisia	Vitamin D supplementation	In patients with acute coronary syndrome, vitamin D supplementation was accompanied by changes in ventricular, biomarker, and regulatory-RNA outcomes; *VDR* variants showed prognostic associations but were not demonstrated to modify treatment efficacy (56). In adults with vitamin D deficiency, *GC* and *CYP2R1* variants were associated with differences in serum 25-hydroxyvitamin D response to supplementation, without assessment of cardiovascular outcomes (98).	(56, 98)
Polygenic, epigenomic, transcriptomic, and multi-omics frameworks- genome-wide interaction scores; CAD and CVD PRS; multivariant GRS; DNA-methylation signatures; transcriptomic profiles; gut metagenomic, metabolomic, and bile-acid features	RCTs and secondary analyses; prospective cohorts; EWAS and GWAS; single-arm interventions; population-based and multi-omics studies; US, UK, Canada, Spain, Israel, Finland and seven Eurpoean countries (Food4Me)	Mediterranean and low-fat diets; dietary fat reduction; weight-loss diets; omega-3; fruit interventions; overall diet quality personalized nutrition	A 1,760-variant score explained part of the variability in LDL-C response to dietary fat reduction (32), whereas large-scale genome-wide analyses generally identified small or unreplicated interaction effects (33). Lifestyle and dietary interventions improved cardiometabolic risk factors largely independently of CAD or CVD PRS with limited or inconsistent interaction evidence (34, 35, 102). *CPT1A* methylation predicted triglyceride and VLDL responses to low-fat weight-loss diets, while Mediterranean-diet and omega-3 studies identified additional diet-related methylation signatures (54, 55, 70). Mediterranean-diet, hydroxytyrosol, and mango interventions produced molecular, microbial, metabolic, or expression changes, but several analyses did not include genotype stratification (10, 29, 51, 61). Host-genetic, dietary microbiome, and metabolomic studies illustrate system-level interactions, although some provide observational or cardiovascular context evidence rather than direct tests of genotype guided dietary treatment (103–107).	(10, 29, 32–35, 51, 54, 55, 61, 70, 71, 74, 102, 107)
Xenobiotic metabolism-*CYP1A2* rs762551	Cross-sectional analysis; Romania	Habitual caffeine intake	Associations of caffeine intake with total cholesterol and fasting glucose varied according to *CYP1A2* rs762551 and concurrent statin or oral-antidiabetic therapy. Because the study was cross-sectional, the findings do not establish genotype-specific effects of caffeine intervention (108).	(108)
Obesity and energy expenditure genes-*FTO* rs1121980 and rs9939609, *ADRB3* rs4994	Multicomponent personalized nutrition RCT and randomized double-blin trial; China and Japan	Personalized nutrition; low dose trans fatty acids	In a multicomponent personalized nutrition intervention, *FTO* rs1121980 modified changes in weight, BMI, waist circumference, total cholesterol, and LDL-C; associated microbiome findings were exploratory (119). in a healthy Japanese adults, supplementation with trans fatty acids at approximately 1% of energy did not significantly alter serum lipids or glycemic measures, and responses were not modified by *FTO* rs9939609 or *ADRB3* rs4994 (126).	(119, 126)

### 4.2 Low-fat and hypocaloric dietary patterns: genotype-dependent cardiovascular responses

Low-fat and hypocaloric dietary patterns are characterized by reductions in total fat intake, energy intake, or both. Nutrigenetic evidence indicates substantial interindividual variability in cardiometabolic responses to these dietary patterns, with genetic background potentially influencing lipid, glycemic, and inflammatory outcomes (32, 82, 112). However, accumulating nutrigenomic evidence demonstrates substantial interindividual variability in response, with genetic background significantly influencing lipid, glycemic, and inflammatory outcomes.

Genome-wide analyses provide strong support for polygenic modulation of lipid responses. In a large, randomized trial of approximately 9,000 women without diabetes, 1,760 SNPs collectively explained variability in LDL-C reduction following a low-fat dietary intervention, highlighting that responsiveness is driven by aggregated genetic architecture rather than single variants (32).

Controlled hypocaloric intervention further reinforces this heterogeneity. In adults with obesity, the apolipoprotein A1 (*APOA1* rs670*)* variant was associated with changes in insulin resistance following both high-fat and low-fat hypocaloric diets and with a greater increase in HDL-C among A-allele carriers assigned to the low-fat diet (82). In the POUNDS LOST randomized trial, dietary fat content modified the associations of lipase C, hepatic type (*LIPC* rs2070895), with 2-year changes in total cholesterol, LDL-C, and HDL-C during energy-restricted weight-loss diets (112). These findings suggest that genetic variation in lipid metabolism pathways may contribute to interindividual differences in metabolic responses to hypocaloric diets.

Personalized nutrition studies provide preliminary translational evidence, although they have not established clear superiority of genotype-guided advice. In the Food4Me trial, personalized advice incorporating dietary, phenotypic, and genotypic information produced greater reductions in saturated-fat intake than standard population advice among *APOE* ε*4* carriers but did not produce significantly greater biomarker improvements or outperform non-gene-based personalized advice (26). In a secondary Food4Me analysis in which the intervention groups were pooled, higher baseline Mediterranean-diet adherence was associated with greater reduction in BMI, waist circumference, and glucose, whereas participants with a lower genetic risk score showed a greater reduction in total cholesterol. Evidence of interaction between the genetic risk score and Mediterranean-diet adherence was limited to changes in total carotenoids and was not observed for BMI or waist circumference (102).

Macronutrient substitution within low-fat diets also interacts with genotype. In the RISCK trial, replacing saturated fat with low-glycemic-index carbohydrates produced greater reductions in total cholesterol and apolipoprotein B among *APOE* ε*4* carriers than among participants with the ε*3/*ε*3* genotype (72).

Collectively, evidence from genome-wide interaction studies, hypocaloric interventions, and nutrigenetic trials indicates that low-fat and energy-restricted diets yield heterogeneous cardiovascular outcomes (Table 2). Genetic variation in lipid transport and energy metabolism pathways significantly modifies responsiveness, suggesting that low-fat strategies may be most effective when tailored to individual genetic profiles rather than universally applied (Figure 5).

**Figure 5:**
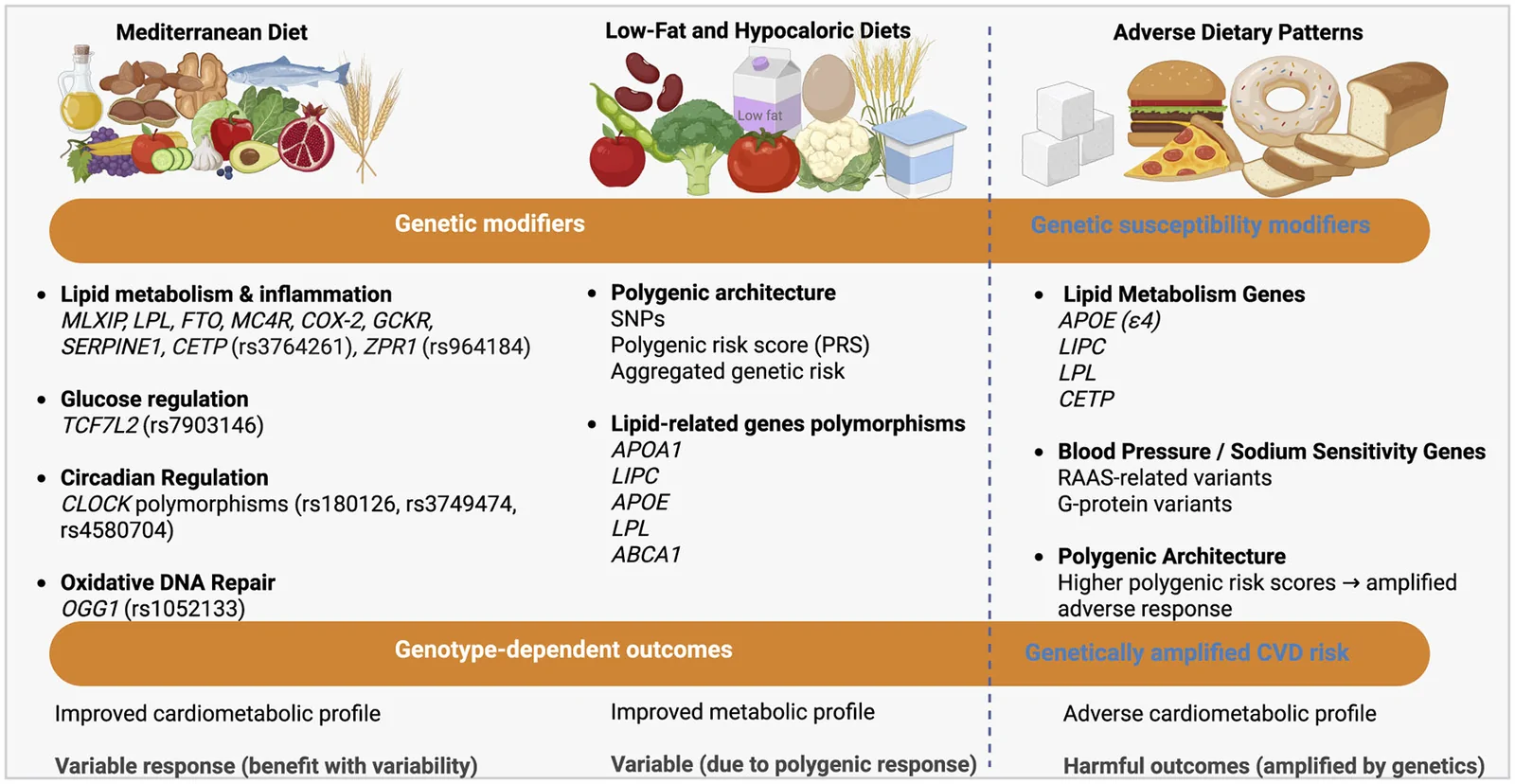
Genotype-dependent cardiovascular responses to dietary patterns. Genetic variation modifies responses to dietary patterns, influencing lipid profiles, inflammation, and cardiovascular risk. Created with BioRender.com.

### 4.3 Adverse dietary patterns and genetic susceptibility

Poor diet quality, characterized by high intake of saturated and trans fats, refined grains, ultra-processed foods, added sugars, and sodium, is associated with increased cardiovascular morbidity and mortality (113). Nutrigenomic evidence suggests that responses to these adverse dietary exposures may vary according to genetic background.

Longitudinal evidence suggests that associations between Western dietary patterns and cardiometabolic risk may vary with aggregate genetic susceptibility and ancestry. In the ARIC cohort, a Western dietary pattern was associated with higher metabolic-syndrome risk among White participants, and associations between dietary patterns and metabolic syndrome varied across genetic-risk score strata and ancestry groups (114). These findings suggest that genetic predisposition may contribute to heterogeneity in the cardiometabolic associations of adverse dietary patterns.

Variants in lipid metabolism genes, including *APOE, LIPC, LPL*, and *CETP*, have been associated with differential lipid responses to diets varying in saturated fat and carbohydrate content (72–74). In a randomized RISCK intervention, replacing saturated fat with low-glycemic-index carbohydrate produced greater reductions in total cholesterol and apolipoprotein B among *APOE* ε*4* carriers than among ε3/ε3 participants (72). Beyond lipid outcomes, the controlled SATgene intervention found that high-fat, high-saturated-fat diets increased C-reactive protein concentrations among *APOE* ε*3/*ε4 participants but not among *APOE* ε*3/*ε*3* participants, although cholesterol responses did not differ significantly by genotype (115).

Adverse dietary patterns also interact with blood pressure-related genes. High sodium intake disproportionately increases hypertension risk in individuals carrying RAAS-related genes or G-protein signaling polymorphisms (37, 41). Longitudinal cohorts further demonstrate that unhealthy diets could amplify inherited CVD risk, whereas healthier patterns attenuate genetic susceptibility (34, 35).

In brief, these findings show that adverse dietary patterns do not affect all individuals equally; genetic background substantially modifies vulnerability, supporting the need for risk-stratified dietary guidance (Table 2).

### 4.4 Personalized nutrition trials and translational evidence

Building on mechanistic insights, randomized trials have evaluated whether genotype-informed dietary interventions improve cardiometabolic outcomes beyond standard recommendations. These studies represent a critical step toward translating gene-diet interaction research into clinical practice.

In the Food4Me trial, personalized advice incorporating diet, phenotype, and genotype produced greater reductions in saturated-fat intake than standard population advice among both *APOE* ε*4* carriers and non-carriers. However, responses did not differ significantly between genotype groups, and genotype-informed advice did not produce greater biomarker improvements or outperform non-gene-based personalized advice (26). Similarly, genotype-guided hypocaloric diets produced larger reductions in triglycerides, VLDL-C, the triglyceride-to-HDL-C ratio, interleukin-6, and tumor necrosis factor-α (74). These preliminary findings suggest potential benefits of genotype-informed dietary advice, although superiority over the standard diet was not consistently demonstrated.

Multi-omics analyses of dietary interventions extend these findings. In the 18-month DIRECT-PLUS trial, integrating host genetic, gut metagenomic, and metabolomic data improved the prediction of individual responses to Mediterranean-diet interventions and identified diet-related changes in microbial and metabolic profiles (61). Separately, transcriptomic analyses from PREDIMED and PREDIMED-Plus reported modest changes in the expression of cholesterol-efflux-related genes following Mediterranean-diet interventions, without genotype stratification (29). These findings illustrate the potential value of molecular measurements for characterizing interindividual dietary responses.

Targeted personalization using vitamin D, omega-3 fatty acids, or polyphenol-rich foods further suggests that variants could influence genotype-dependent benefits in *VDR, FADS1, FADS2*, and antioxidant-related genes (56, 67, 90).

Although reported benefits are generally incremental and largely involve intermediate outcomes, such as lipid profiles, inflammatory markers, dietary behavior, or molecular signatures, interpretation of evidence requires caution.

Single-SNP analyses may underestimate genetic effects in complex polygenic phenotypes (116), while the clinical application of polygenic-risk information raises ethical, legal, social, and interpretive concerns (117). Accordingly, routine incorporation of genetic information into cardiovascular nutrition counseling remains premature pending stronger evidence of reproducibility, clinical utility, and implementation feasibility.

Overall, personalized nutrition trials provide emerging translational evidence that gene-informed dietary strategies may contribute to CVD prevention and management. However, their clinical value beyond established phenotype- and behavior-based personalization remains uncertain, and genotype-guided nutrition should currently be regarded as an investigational rather than routine component of cardiovascular care (Table 2).

## 5 Clinical application and translation to personalized nutrition

### 5.1 Current clinical applications of nutrigenomics and nutrigenetics

Advances in nutrigenetics and nutrigenomics are increasingly informing personalized dietary strategies for cardiovascular disease prevention and management (118). Although most clinical guidelines remain population-based, genomic studies continue to investigate sources of interindividual variability in cardiometabolic responses to diet.

In research and specialized clinical settings, nutrigenetic testing has been investigated as a means of characterizing interindividual variation in lipid, glycemic, and other cardiometabolic responses (118). In a 12-week randomized trial of adults with overweight or obesity, *FTO rs1121980* genotype modified changes in weight, BMI, waist circumference, total cholesterol, and LDL-C following a personalized nutrition intervention, with gut-microbiota differences explored as a potential contributor (119). Human intervention studies have reported exposure-specific associations between genetic variants and dietary responses. In the RISCK trial, *APOE* ε*4* carriers showed greater reductions in total cholesterol and apolipoprotein B when dietary saturated fat was replaced with low-glycemic-index carbohydrate (72). In *post hoc* analysis of OMEGA-REMODEL, *FADS2* was associated with differential cardiac-remodeling and biomarker responses to high-dose omega-3 fatty acid supplementation after acute myocardial infarction (67). In adults with type 2 diabetes, *FADS1* was associated with lipid response during interventions using sesame, canola, and sesame-canola oils (120). Genotype-stratified differences in triglyceride-rich lipoprotein profiles following low-fat and Mediterranean diets were also reported for *TCF7L2*, although the formal genotype-by-diet interaction was not statistically significant (64). In PREDIMED, the association between *CLOCK* and incident type 2 diabetes was stronger among participants assigned to Mediterranean-diet interventions than among those assigned to a control diet, with borderline evidence of genotype-by-intervention interaction (25).

Beyond single-variant approaches, PRS have been investigated as potential modifiers of dietary and lifestyle responses rather than as established tools for assigning dietary interventions. Across intervention, observational, and secondary analyses, evidence of PRS-diet interaction was limited and inconsistent, while benefits from healthier dietary or lifestyle patterns were generally observed across genetic-risk strata (34, 35, 102). Current evidence therefore does not establish superior outcomes from PRS-guided dietary prescribing.

However, clinical translation of PRS remains limited. A recent European Society of Cardiology consensus statement emphasizes that PRS should complement and not replace established cardiovascular risk assessment tools (121). The statement notes that PRS may be most useful in individuals at borderline or intermediate risk, where genetic information could refine risk classification, while acknowledging that PRS generally provides incremental predictive value rather than stand-alone clinical utility, with ancestry-specific limitations and limited evidence for improved clinical outcomes following PRS disclosure (121).

Nutrigenomics further extends clinical applicability by identifying molecular signatures that reflect biological response to dietary interventions (122). Transcriptomic and epigenomic studies show that Mediterranean, low-fat, and energy-restricted diets induce changes in gene expression related to lipid transport, oxidative stress defense, inflammation, and endothelial function (29, 123, 124). These molecular adaptations may serve as early biomarkers of dietary responsiveness before changes in clinical endpoints are evident.

Personalized nutrition interventions integrating genetic, phenotypic, and dietary data have shown improvements in lipid profiles, inflammatory markers, and metabolic outcomes compared with generalized dietary advice in some studies (10, 26, 29, 49, 56, 67, 74, 90). However, reported benefits are generally incremental, study designs are heterogeneous, and many outcomes remain intermediate cardiometabolic markers rather than hard cardiovascular endpoints.

Despite these advances, routine clinical implementation remains limited and is currently most feasible in specialized or research-informed settings. Nevertheless, accumulating evidence supports the integration of genetic and molecular insights into dietary counseling as an adjunct to established cardiovascular prevention frameworks (Figure 6).

**Figure 6:**
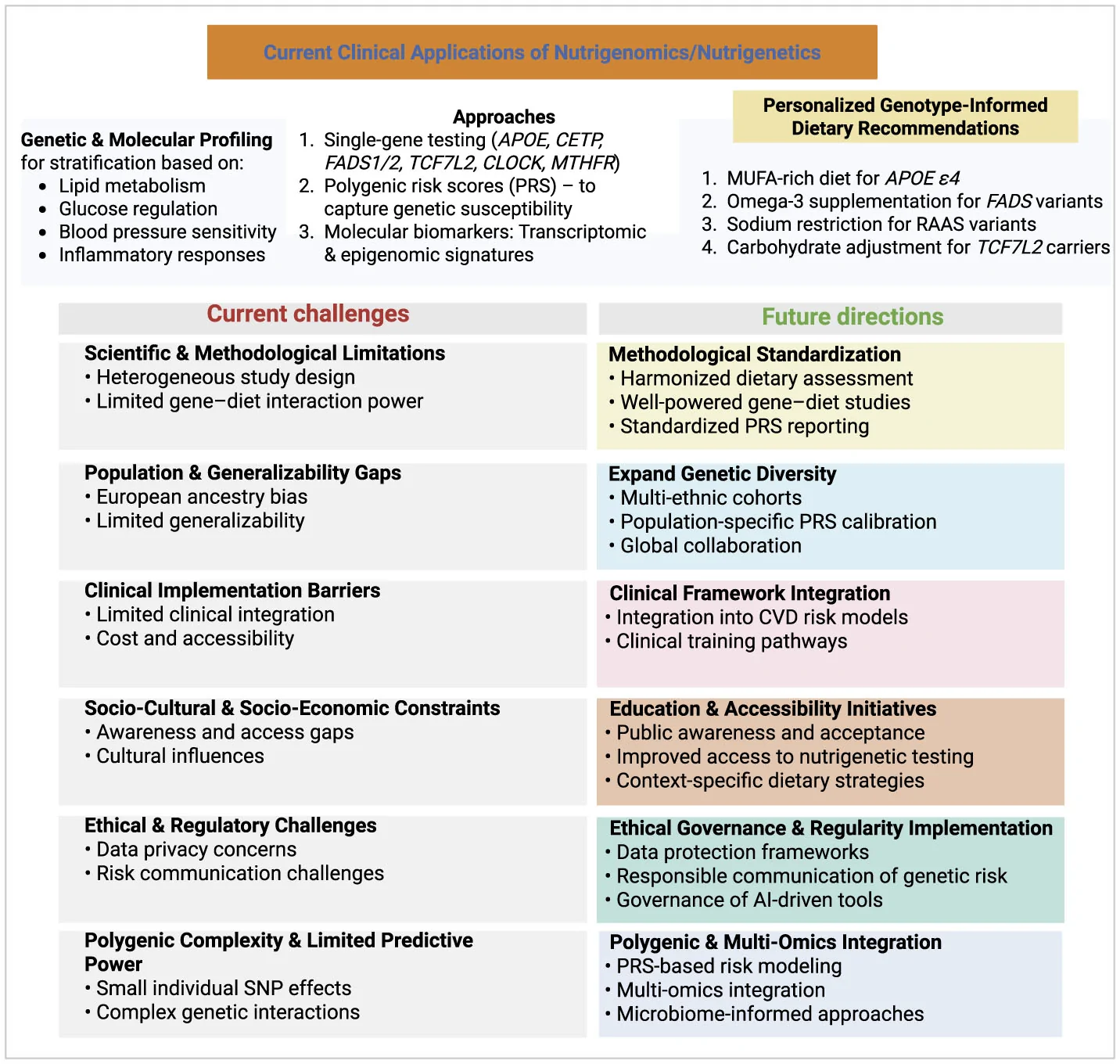
Current applications, challenges and future directions in clinical implementation of nutrigenomics in CVD. Created with BioRender.com.

### 5.2 Limitations and challenges in applying nutrigenomics to CVD care

Despite expanding evidence supporting gene-diet interactions in cardiovascular disease (CVD), several limitations constrain interpretation, generalizability, and clinical translation. The strength of evidence varies substantially across nutrigenetic and nutrigenomic findings. Evidence is strongest when genotype-dependent dietary responses are supported by randomized controlled trials, controlled feeding studies, prospective cohorts, Mendelian randomization analyses, or genome-wide interaction studies with independent replication. In contrast, many reported gene-diet interactions are derived from observational cohorts, secondary analyses, candidate-gene studies, or relatively small intervention studies, which are more vulnerable to residual confounding, population stratification, multiple-testing burden, and limited statistical power. Replication across independent cohorts remains inconsistent for several variants, and findings may differ according to ancestry, baseline cardiometabolic risk, dietary assessment method, intervention duration, and outcome definition. Therefore, statistically significant associations should not automatically be interpreted as clinically actionable. For precision cardiovascular nutrition, clinical relevance depends on whether genotype-guided recommendations produce meaningful improvements in validated cardiometabolic endpoints, such as LDL-C, triglycerides, blood pressure, glycemic control, inflammatory markers, or cardiovascular events, beyond standard phenotype-based dietary counseling. At present, many findings support biological plausibility and risk stratification, but fewer have demonstrated sufficient effect size, reproducibility, and clinical utility to justify routine implementation. Therefore, throughout this review, observational gene-diet findings are interpreted as associative unless supported by randomized intervention data, controlled feeding evidence, Mendelian randomization, or replication across independent cohorts.

A major concern is limited population diversity. Most nutrigenomic studies have been conducted in populations from Europe and North America, including countries such as Spain, the United Kingdom, the United States, Canada, and multi-country European cohorts, with comparatively fewer studies from Asia (China, Japan, Taiwan, Korea), the Middle East, North Africa, and Latin America. Given differences in allele frequencies, linkage disequilibrium structure, dietary exposures, and baseline cardiometabolic risk, findings from predominantly European cohorts may not be directly transferable. This restricts the external validity of genetic risk scores and gene-diet interaction models and may contribute to inconsistent replication.

Another important limitation is the inconsistent and often population-specific replication of gene-diet interactions, alongside small-to-moderately sized study populations, particularly in interaction analyses. Many findings are derived from single cohorts or population-specific analyses and have not been consistently reproduced in independent populations. Small or moderately sized samples may limit statistical power to detect true interactions and increase the risk of false-positive findings. As a result, caution is warranted when interpreting reported associations, and broad generalizations or causal assertions should be made carefully, especially when evidence is based on isolated or non-replicated studies.

These concerns are consistent with recent clinical consensus statements, which emphasize that PRS currently provide incremental rather than standalone predictive value and should be cautiously integrated within established cardiovascular risk frameworks (121). Similarly, evidence from population-based studies suggests that genetic susceptibility alone provides limited predictive power when considered independently of conventional risk factors. In a case-control study nested within the Greek cohort of the European Prospective Investigation into Cancer and Nutrition (EPIC), a genetic predisposition score composed of multiple polymorphisms was associated with coronary infarction primarily when combined with established risk factors such as hypertension, dyslipidemia, smoking, and poor adherence to the Mediterranean diet. Individual polymorphisms showed limited predictive ability, highlighting that traditional cardiometabolic and lifestyle determinants remain dominant contributors to cardiovascular risk (125).

Methodological heterogeneity further complicates synthesis. Study designs range from randomized controlled trials to observational cohorts, genome-wide interaction analyses, and Mendelian randomization studies, each with distinct assumptions and biases. For example, a randomized controlled trial in healthy Japanese adults found that supplementation with trans fatty acids equivalent to 1% of total energy intake did not significantly alter serum cholesterol concentrations regardless of obesity-related genotypes, including fat mass and obesity associated gene (*FTO*) and β3-adrenergic receptor variants, suggesting that genetic variation does not always modify metabolic responses to dietary exposures (126). Dietary assessment methods, including food frequency questionnaires, recalls, biomarkers, and controlled feeding, vary in precision and susceptibility to misclassification. Genetic analyses span candidate-gene analyses, PRS analyses, and transcriptomic approaches, limiting comparability across studies.

Design-related limitations also persist. Many studies are secondary or not adequately powered for gene-diet interaction analyses. Short intervention duration may fail to capture long-term adaptations, while cross-sectional designs limit causal inference. Residual confounding from medication use, baseline metabolic status, physical activity, and sleep patterns remains possible.

In addition, several controlled dietary interventions report null gene-diet interaction effects. For example, a randomized crossover trial evaluating docosahexaenoic acid (DHA) supplementation demonstrated significant reductions in circulating triglycerides of approximately 24%, yet no significant associations were observed between lipid responses and multiple single-nucleotide polymorphisms involved in lipid metabolism pathways (127). These findings indicate that beneficial cardiometabolic responses to certain dietary interventions may occur independently of genetic variation, suggesting that not all diet-induced metabolic improvements are strongly genotype dependent.

Beyond methodological issues, several health-system and implementation barriers must be addressed before nutrigenomics can be incorporated into routine cardiovascular care. Socio-economic and cultural determinants shape dietary behavior and adherence, yet these factors are rarely incorporated into nutrigenomic models. Consumer attitudes, privacy concerns, cost barriers, skepticism toward genetic testing, and limited access to trained professionals may further restrict uptake. The cost-effectiveness of nutrigenetic testing remains uncertain, since demonstrated clinical benefits are generally incremental, largely confined to intermediate outcomes, and not yet established for long-term cardiovascular outcomes. Reimbursement is another major barrier, as inconsistent coverage for genetic testing, interpretation, and dietitian-led counseling may restrict access to individuals who can pay out of pocket, potentially widening existing health disparities. Clinician education is also essential, as cardiologists, primary care physicians, dietitians, and other health professionals require training to interpret genetic reports, evaluate the strength of evidence for specific gene-diet interactions, recognize ancestry-related limitations of polygenic risk scores, and communicate genetic risk without overstating clinical certainty. Importantly, genotype-based feedback does not consistently outperform phenotype- or behavior-based personalization; therefore, genetic reports should not be interpreted as stand-alone dietary prescriptions, but rather integrated with established conventional cardiovascular risk assessment, family history, biochemical markers, medication use, dietary patterns, and patient preferences. Regulatory oversight and standardized reporting are also needed to ensure analytical and clinical validity, transparent variant interpretation, data privacy, responsible communication of risk, and appropriate governance as genetic and multi-omics tools move toward clinical application (121, 128).

Collectively, while nutrigenomics provides valuable mechanistic insights into interindividual variability, current evidence does not yet support widespread clinical implementation. Greater population diversity, methodological harmonization, longer-term trials, and integration of socio-cultural determinants will be essential to advance nutrigenomics from discovery science to equitable CVD care.

### 5.3 Future perspectives and precision-nutrition frameworks

Nutrigenomics and nutrigenetics are informing research directions in cardiovascular prevention by moving beyond single-variant models toward integrated, systems-based precision nutrition frameworks. Future progress will depend on combining molecular resolution with clinical feasibility, population-level relevance, and rigorous validation in diverse clinical settings.

A central direction involves polygenic and multi-layered models that integrate PRS with epigenetic markers, transcriptomic signatures, metabolomics, microbiome profiles, and phenotypic data to predict dietary responsiveness. Genome-wide interaction studies suggest that aggregated genetic risk better captures variability in lipid metabolism, glycemic regulation, and blood pressure response than individual variants alone, potentially refining dietary recommendations. However, these models require external validation across diverse ancestry groups and clinical populations before they can be translated into routine cardiovascular nutrition practice.

Integration of multi-omics and microbiome data represents another major advance. Diet-induced changes in DNA methylation, non-coding RNAs, gut microbial composition, and circulating metabolites may mediate cardiometabolic responses to fats, carbohydrates, fiber, and polyphenols. Large-scale precision nutrition initiatives integrating genomic, microbiome, metabolic, continuous glucose monitoring, and dietary data have demonstrated substantial interindividual variability in postprandial metabolic responses (129, 131). Such approaches may enable adaptive nutrition strategies responsive to both inherited risk and modifiable molecular states.

Technological innovation may further support translation. Zeevi et al. developed a machine learning model using clinical, dietary, physical-activity, and gut-microbiome data from 800 participants to predict individualized postprandial glycemic responses to real-life meals; its predictions were validated in an independent cohort of 100 participants (130). This study demonstrated prediction of short-term glycemic responses but did not evaluate cardiovascular outcomes. Therefore, the use of AI to integrate multi-omics data and support cardiovascular nutrition decisions remains a potential future application requiring prospective validation in diverse populations using clinically meaningful cardiovascular endpoints.

Future research should also prioritize longitudinal, multi-ethnic cohort studies with repeated dietary, genetic, multi-omics, microbiome, and cardiometabolic measurements. Such studies are needed to determine whether molecular signatures of dietary response remain stable over time and whether they predict durable improvements in cardiovascular risk. In parallel, standardized reporting guidelines for nutrigenetic and nutrigenomic studies are required to improve comparability across studies. These guidelines should include clear reporting of dietary assessment methods, ancestry and population structure, variant selection, PRS construction, validation cohorts, effect sizes, null findings, and clinical actionability.

Implementation science approaches will be essential to evaluate how precision nutrition can be incorporated into real-world cardiovascular care. Future studies should assess feasibility, acceptability, cost-effectiveness, reimbursement pathways, clinician education, workflow integration, patient understanding, and equity of access. Prospective clinical trials are particularly needed to determine whether genotype-guided nutritional interventions improve validated cardiometabolic endpoints or cardiovascular outcomes beyond standard phenotype-based dietary counseling. These trials should be adequately powered, include diverse populations, use clinically meaningful outcomes, and compare genotype-guided advice with current guideline-based dietary recommendations.

From a public health perspective, equitable implementation requires intentional inclusion of diverse populations and incorporation of social, cultural, economic, and environmental determinants alongside molecular data. Precision nutrition must extend beyond genomic profiling to encompass real-world contexts, including food access, affordability, health literacy, cultural dietary practices, and healthcare infrastructure, to avoid widening health disparities (128).

Ethical, regulatory, and clinical implementation considerations are equally critical. Clear standards for genetic data governance, privacy protection, analytical validity, clinical validity, and responsible communication of genetic risk are needed. As AI-driven models expand, robust oversight and clinician education will be essential to prevent overreliance on algorithmic outputs and to ensure that genetic and multi-omics information is integrated within holistic lifestyle counseling rather than used as a stand-alone dietary prescription (128).

Overall, advancing precision cardiovascular nutrition will require multidisciplinary collaboration, rigorous validation of gene-diet interactions, inclusive longitudinal cohorts, standardized reporting frameworks, implementation science approaches, and prospective clinical trials evaluating genotype-guided dietary interventions. These efforts should aim to integrate molecular insight with clinical feasibility, cost-effectiveness, behavioral counseling, and social and environmental determinants of health, ensuring that precision nutrition becomes clinically actionable, equitable, and complementary to established cardiovascular prevention strategies.

## 6 Conclusion

CVD arises from dynamic interactions between diet, genetic variation, and molecular regulation rather than from isolated risk factors alone. Evidence from nutrigenomics, nutrigenetics, and multi-omics research suggests that dietary exposures modulate gene expression, epigenetic remodeling, and metabolic pathways governing lipid metabolism, glucose regulation, inflammation, and blood pressure. These mechanisms provide a biological explanation for the substantial interindividual variability observed in cardiometabolic responses to dietary interventions.

Randomized trials, cohort studies, and genome-wide analyses collectively suggest that genetic variation across key cardiometabolic pathways may modify responsiveness to dietary fats, carbohydrates, sodium, and plant-derived bioactives. Polygenic and system-level approaches further indicate that cumulative genetic susceptibility may contribute to variability in dietary response. In the future, validated genetic information may help refine dietary recommendations for selected individuals, but it should complement rather than replace established population-level dietary guidance.

Clinical implementation remains limited by methodological heterogeneity, insufficient population diversity, limited external validation, and the fact that many reported benefits are incremental or restricted to intermediate cardiometabolic outcomes. Nevertheless, the integration of genomics, epigenomics, transcriptomics, metabolomics, and microbiome science is expanding understanding of interindividual variation in cardiovascular nutrition. Advancing precision cardiovascular nutrition will require rigorous validation, inclusive research, and clinically meaningful endpoints, and frameworks that are feasible and equitable in practice. Gene-diet interactions, therefore, remain an active area of research with potential relevance to the future refinement of CVD prevention and management.
